# Potential prognosis and immunotherapy predictor TFAP2A in pan-cancer

**DOI:** 10.18632/aging.205225

**Published:** 2024-01-23

**Authors:** Chenxi Niu, Haixuan Wen, Shutong Wang, Guang Shu, Maonan Wang, Hanxi Yi, Ke Guo, Qiong Pan, Gang Yin

**Affiliations:** 1 Department of Pathology, Xiangya Hospital, School of Basic Medical Sciences, Central South University, Changsha, China; 2 Xiangya Medical School, Central South University, Changsha, China; 3 Department of Neurology, The Third Xiangya Hospital of Central South University, Changsha, China; 4 Department of Obstetrics and Gynecology, The Third Xiangya Hospital of Central South University, Changsha, China; 5 China-Africa Research Center of Infectious Diseases, School of Basic Medical Sciences, Central South University, Changsha, China; 6 National Clinical Research Center for Geriatric Disorders, Xiangya Hospital, Central South University, Changsha, China

**Keywords:** TFAP2A, pan-cancer, tumor microenvironment, immunotherapy, PD-L1

## Abstract

Background: TFAP2A is critical in regulating the expression of various genes, affecting various biological processes and driving tumorigenesis and tumor development. However, the significance of TFAP2A in carcinogenesis processes remains obscure.

Methods: In our study, we explored multiple databases including TCGA, GTEx, HPA, cBioPortal, TCIA, and other well-established databases for further analysis to expound TFAP2A expression, genetic alternations, and their relationship with the prognosis and cellular signaling network alternations. GO term and KEGG pathway enrichment analysis as well as GSEA were conducted to examine the common functions of TFAP2A. RT-qPCR, Western Blot and Dual Luciferase Reporter assay were employed to perform experimental validation.

Results: TFAP2A mRNA expression level was upregulated and its genetic alternations were frequently present in most cancer types. The enrichment analysis results prompted us to investigate the changes in the tumor immune microenvironment further. We discovered that the expression of TFAP2A was significantly associated with the expression of immune checkpoint genes, immune subtypes, ESTIMATE scores, tumor-infiltrating immune cells, and the possible role of TFAP2A in predicting immunotherapy efficacy. In addition, high TFAP2A expression significantly correlated with several ICP genes, and promoted the expression of PD-L1 on mRNA and protein levels through regulating its expression at the transcriptional level. TFAP2A protein level was upregulated in fresh colon tumor tissue samples compared to that in the adjacent normal tissues, which essentially positively correlated with the expression of PD-L1.

Conclusions: Our study suggests that targeting TFAP2A may provide a novel and effective strategy for cancer treatment.

## INTRODUCTION

Transcription factor activating enhancer binding protein 2 (TFAP2), a vital class of transcription factors whose family members include five different paralogs in humans and mice: Tfap2a to Tfap2e, encoding proteins α, β, γ, δ, and ε respectively [1, 2]. All AP-2 proteins possess a highly conserved helix-span-helix dimerization motif at the carboxyl terminus, followed by a central motif region with a less conserved proline-rich and the glutamine-rich region at the amino terminus [3]. These proteins can form heterodimers and homodimers, which are able to regulate the specific expression of various downstream genes through binding to the functional element on their promoters, thereby influencing various biological processes such as embryonic development, cell proliferation, cell differentiation, cell migration, and cell apoptosis [4–6].

TFAP2A, a member of TFs AP-2 family, binds to CG-rich sequences through a DNA-binding domain to regulate the transcription of downstream genes [7]. TFAP2A is reported to be involved in vertebrate early embryogenesis of tissues undergoing morphogenetic transformation, especially in the differentiation and development of facial prominences, neural crest, kidney, and eye [3, 6, 8–10]. Mutations in TFAP2A lead to branchio-oculo-facial syndrome, a kind of disease that is characterized by skin defects, ocular anomalies, and facial clefting [11]. Nowadays, studies have shown that TFAP2A plays essential roles in various tumor-associated biologic processes, including cell cycle, cell apoptosis, and Epithelial-mesenchymal transition (EMT) [12–14]. Because of the differences across intra-tumoral heterogeneity of human cancers, TFAP2A has paradoxical effects on different kinds of carcinomas. For instance, in Lung adenocarcinoma (LUAD), TFAP2A promotes EMT indirectly via transcriptionally overexpressing KRT16. Moreover, the binding of TFAP2α at certain Hoxa gene loci promotes the development of acute myeloid leukemia [15]. And TFAP2A accelerates drug resistance by promoting angiogenesis in lung cancer [16]. However, it can also act as a tumor suppressor in glioma, colon cancer, melanoma, clear cell renal cell carcinoma, and so on [17–20].

Although several studies have reported that TFAP2A promoted cancer progression by regulating different pathways, the common function of TFAP2A in pan-cancer has rarely been analyzed systematically. In this study, we first analyzed the expression of TFAP2A, and its prognosis values, genomic alternations in pan-cancer. Next, we explored its correlations with co-expressed genes and differential genes in various cancer types. By integrating the results, we found the potential common roles of TFAP2A in pan-cancer were related to tumor immunity. Following that, we explored the relationship between TFAP2A expression with Immune checkpoint (ICP) genes, immune subtypes, immune cell infiltrations, immunotherapy efficacy, and PD-L1 expression in cancer cell lines to unravel the role of TFAP2A from the perspective of tumor immunity. Taken together, this study highlighted the potential role of TFAP2A in pan-cancer, hence offering a novel and promising insight into prognosis and immunotherapy.

## MATERIALS AND METHODS

### Data handling and TFAP2A expression analysis

TFAP2A expression TOIL TPM data of pan-cancer tissues were obtained from TCGA via UCSC Xena Browser and GTEx v.7 TPM data of normal tissues were accessed from the GTEx Portal [21]. All analyses of expression data were performed using log2(TPM+1) values. Data exploration and processing, statistical analysis, and visualization were performed using Strawberry Perl scripts software (Version: 5.32.1.1) and R (version: 3.6.3).

TIMER2 database was also employed for cross-verification of expression data. TFAP2A mRNA and protein expression in cancer cell lines was accessed using CCLE [22].

### Survival prognosis analysis

Level 3 RNASeq HTSeq-FPKM files for cancer patients were downloaded from TCGA. Filtering was made to exclude the data of normal samples or samples with missing clinical information and further processing was to determine the ideal point to divide groups using the R-package “survminer”. Kaplan–Meier plotter analysis and Cox proportional hazard models were used to identify the relationship between TFAP2A mRNA expression and survival outcomes indexes including Overall survival (OS), Disease specific survival (DSS) and Progression-free interval (PFI). Hazard ratios (HR) with corresponding 95% Confidence intervals (CIs) and log-rank P-values were calculated. Cox regression multivariable analysis was used to build the nomogram prognostic model to predict the probability of a patient event. The R-packages “survival”, “survminer”, “ggplot2” and “rms” were employed to perform statistical analyses and visualization.

### Genomic alterations in pan-cancer

TFAP2A genomic alterations analysis based on the data from TCGA PanCancer Atlas Studies were conducted by using cBioPortal database [23]. Genetic alternations in single cancer study, alternations summary of multiple cancers, mutation types and sites of TFAP2A were obtained from “Cancer Type Summary”, “OncoPrint” and “Mutations” modules, respectively. Using “Plots” module to investigate the relationship between Copy number variation (CNV) and TFAP2A mRNA expression. Based on whether the genetic alternation occurred, TFAP2A related alternation genes were identified via “Comparison” module.

### TFAP2A gene correlation analysis in pan-cancer

To assess the potential functional significance of TFAP2A, we performed gene correlation analysis by analyzing RNASeq data from TCGA (excluding normal samples data and transferring FPKM values to the log2 of TPM). Ensembl 101 was used for gene annotations. We divided samples into two groups, separated by the median value of TFAP2A mRNA expression. To explore a more general association between TFAP2A and cancer, we used Pearson’s correlation coefficients greater than 0.3 and Pearson P-values less than 0.05 as the cutoff to select preliminary gene candidates. Then occurrence frequencies of genes in 33 cancer types meeting the above conditions were determined for Gene ontology (GO) term and Kyoto Encyclopedia of Genes and Genomes (KEGG) pathway enrichment analysis. IDs conversion was done using R-package “org.Hs.eg.db”, and for enrichment analysis was “clusterProfiler”. The R-packages “stat”, “DESeq2”, and “ggplot2” were used to perform statistical analyses and visualization.

### Gene set enrichment analysis

We evaluated the differential gene expression in low and high TFAP2A expression samples, which was implemented by the “DESeq2” R-package. To further explore the biological functions of TFAP2A in cancer, we performed a GSEA using the MSigDB C2 canonical pathways collection and found the common pathways in various cancer types. TFAP2A gene alternation-related genes were analyzed in the same way. The R-Packages “clusterProfiler” was used for Gene set enrichment analysis (GSEA) analysis. The enrichment with False discovery rate (FDR, q-value) less than 0.25 and p-value less than 0.05 was considered as a significant enrichment.

### Association analysis of TFAP2A expression with the expression of ICP genes and immune subtypes

To explore the correlation between the expression of TFAP2A and cancer immunity, we did a correlation analysis of TFAP2A with ICP genes collected from previous literature using TIMER2 and visualized the results using “ggplot2” R-package [24, 25]. Analysis of TFAP2A expression with immune subtypes was explored via the TSDIB database [26].

### Analysis of TFAP2A expression with tumor immune microenvironment

To infer the composition of the Tumor microenvironment (TME), we calculated the Immune Score, Stromal Score, and ESTIMATE Score through the R-package “estimate”. Single sample GSEA (ssGSEA) computed using the “GSVA” R-package, was performed to investigate immune cell infiltration signatures in TME. ssGSEA utilized the specific markers of various immune cells as a gene set to calculate their enrichment score based on a previous study [27]. Correlation analyses were estimated using Spearman's rho.

### Correlation of TFAP2A expression with immunotherapy

To evaluate the effect of TFAP2A expression on predicting immunotherapy efficacy, we extracted the calculated Immunotherapy Scores of PD-1 or CTLA4 blockade treatment from TCIA (http://tcia.at/) [28]. We separated samples into two groups according to TFAP2A expression and integrated them with immunotherapy scores data using the R-package “limma”. We used the R-package “ggpubr” for results visualization.

### Cell lines and cell culture and plasmid transfection

The breast invasive carcinoma (BRCA) cell line MCF-7 was kindly provided by Chief Physician Jinhui Hu. The COAD cell line Caco2 were kindly provided by Professor Wancai Yang. MCF-7 cells were cultured in DMEM (BI) and Caco2 cells were cultured in RPMI 1640 (BI), and both media were replenished with 10% FBS (BI). All cells were cultured at 37 degrees Celsius in the presence of 5% CO_2_.

5 × 10^5^ cells were seeded in a 6-well plate and transient transfected with 1.5 μg pEGFP-N1 or pEGFP-N1-TFAP2A using lipofectamine 2000 (Thermo Fisher Scientific, USA) according to the manufacturer instructions. These cells from each group were cultured for another 48 h and harvested for RNA or proteins extraction.

### RNA extraction and RT-qPCR analysis

Cells total RNA was extracted with TRIzol reagent (Vazyme, Nanjing, China). 1 ug of RNA was reverse transcribed to cDNA using the GoScript Reverse Transcription System (Promega, USA). Reverse transcription-quantitative polymerase chain reaction (RT-qPCR) was carried out by the Applied Biosystems 7500 Real-Time PCR System using the GoTaq RT-qPCR Master Mix (Promega, A6001). We chose GAPDH to normalize TFAP2A and PD-L1 expression levels. Relative gene expression was calculated using the 2^-ΔΔCt^ method. All the sequences of qPCR primers were shown in Supplementary Table 1.

### Western blot analysis

Cells were harvested and immediately lysed by RIPA strong lysis buffer (Beyotime, China) containing protease inhibitors and phosphatase inhibitors: 1% PMSF (Roche, Mannheim, Germany) and 2% PIC (Roche, Switzerland). After centrifugating for 20 min, protein concentration in the supernatant was collected in tubes and determined using a Pierce™ BCA protein assay kit (Thermo Fisher Scientific, USA). The supernatant was then mixed with protein loading buffer (NCM Biotech) and boiled at 100° C for 5 min. Equal amounts of proteins (10 μg) were electrophoresed and separated by SDS-PAGE gels (Bio-Rad, USA), transferred onto PVDF membranes (Immobilon®-P), and blocked with 5% BSA solution at room temperature. Then the membranes were incubated overnight at 4° C with indicated primary antibodies. Followed by washing membranes three times and incubating with secondary antibodies for 2 h at room temperature, and visualized by the NcmECL Ultra (A+B) chemiluminescence reagent (NCM Biotech) and exposed using a chemiluminescence imaging system (SAGECREATION MiniChemi 610), GAPDH was used as an internal control. Anti-rabbit TFAP2A antibody was purchased from Abclonal (A0416, 1:800), anti-rabbit PD-L1 antibody was purchased from Proteintech (66248-1-Ig, 1:3000), anti-mouse GAPDH antibody was purchased from Utibody (UM4002, 1:2000).

### Dual luciferase reporter assay

Briefly, HEK-293T cells were seeded in the 24-well plate and then cotransfected with plasmids: TFAP2A OE plasmid, Renilla luciferase plasmid along with PD-L1 promoter (-2400 to +100 bp). The luciferase activity was measured for at least three independent experiments by using the Dual-Glo Luciferase Kit (Promega) after transfection for 24 h. Firefly luciferase activity in each sample was calculated by normalization to Renilla activity. According to the binding sites of TFAP2A on PD-L1 promoter predicted by JASPAR (https://jaspar.genereg.net/), four truncated PD-L1 promoter were constructed (-1900 to +100, -1400 to +100, -900 to +100, and -400 to +100 bp) to identify a specific binding site. The five PD-L1 promoter region sequences can be found in Supplementary Table 2.

### Tissues protein extraction

A total of 4 pairs of fresh colon tissue samples and adjacent normal colon tissue samples were obtained by surgical resection, which were acquired from The Third Xiangya Hospital of Central South University (Changsha, China).

## RESULTS

### Differential expression of TFAP2A in pan-cancer and normal tissues

**Figure 1:**
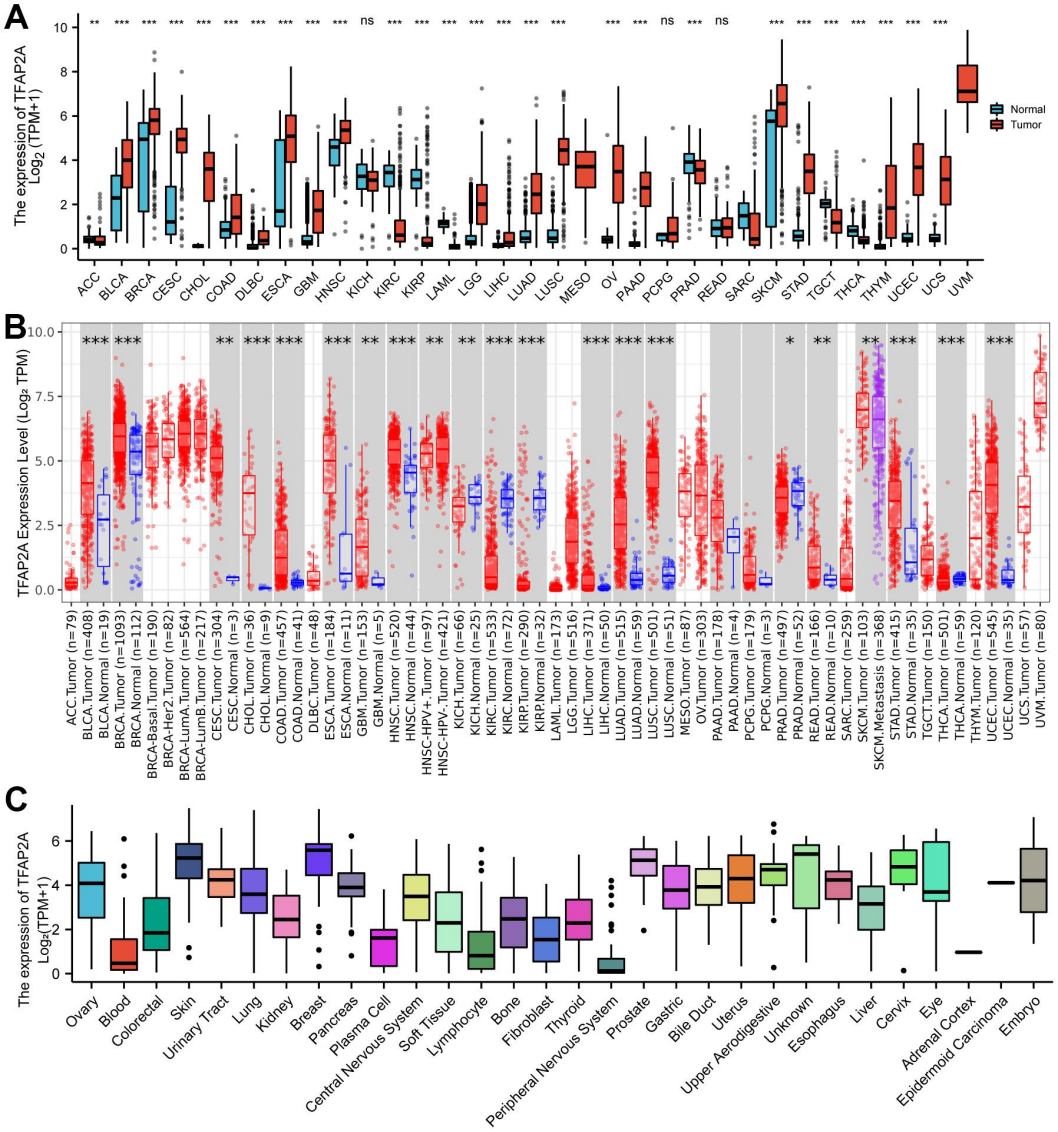
**Different expression of TFAP2A.** (**A**) Different Expression of TFAP2A. (**A**) Differential TFAP2A mRNA expression between unpaired TCGA cancers and GTEX normal tissues; Red columns: cancer samples; Blue columns: normal samples; (**B**) The TFAP2A mRNA expression in 33 cancer types from TIMER database; (**C**) TFAP2A mRNA expression in different cancer cell lines from CCLE database; *p < 0.05, **p < 0.01, and ***p < 0.001.

To investigate the expression of TFAP2A in human pan-cancer, the mRNA levels of TFAP2A were examined based on the TCGA and GTEX databases. The results indicated that TFAP2A mRNA levels not only in non-paired samples but also in paired samples were higher in most human tumors compared to the matched normal tissues (Figure 1A and Supplementary Figure 1B). On the contrary, in the other small fraction of cancer types, TFAP2A mRNA expression was significantly lower, including KIRC, and KIRP. The mRNA expression of TFAP2A were also searched in human pan-cancer by using the TIMER database, which showed that TFAP2A expression was upregulated in Bladder urothelial carcinoma (BLCA), BRCA, Cervical squamous cell carcinoma and endocervical adenocarcinoma (CESC), Cholangiocarcinoma (CHOL), Colon adenocarcinoma (COAD), Esophageal carcinoma (ESCA), Glioblastoma multiforme (GBM), Head and neck squamous cell carcinoma (HNSC), HNSC-HPV, Kidney chromophobe (KICH), Kidney renal clear cell carcinoma (KIRC), Kidney renal papillary cell carcinoma (KIRP), Liver hepatocellular carcinoma (LIHC), LUAD, Lung squamous cell carcinoma (LUSC), Prostate adenocarcinoma (PRAD), Rectum adenocarcinoma (READ), Skin cutaneous melanoma (SKCM), Stomach adenocarcinoma (STAD), Thyroid carcinoma (THCA), Uterine corpus endometrial carcinoma (UCEC) than the control groups (Figure 1B).

Furthermore, TFAP2A mRNA and protein levels in tissue-derived cancer cell lines were examined according to the CCLE database. TFAP2A expression was tissue-specific in various cancer cell lines, especially overexpressed in the breast, prostate, skin in mRNA levels (Figure 1C), and upper in aerodigestive, uterus, esophagus in protein levels (Supplementary Figure 1C). Similar to its expression abundances in tumor tissues, TFAP2A mRNA expression also showed low tissue specificity (Supplementary Figure 1A). Additionally, we analyzed the IHC images provided by HPA database. As shown in Supplementary Figure 1D, the protein level of TFAP2A was significantly higher in BLCA, BRCA, CESC, and SKCM than in corresponding normal tissues (Supplementary Figure 1D). Taken together, TFAP2A has higher expression levels in most types of cancer. Besides, we conducted Western blot to check the constitutive expression of TFAP2A in normal ovarian epithelial cell line FE25 and ovarian cancer cell lines such as 8910, OVCAR3, A2780, and SKOV3 (Supplementary Figure 1E). The expression of TFAP2A was higher in ovarian cancer cell lines than in FE25, which was consistent with the predicted results above.

### The co-relationship between TFAP2A expression and prognosis

**Figure 2:**
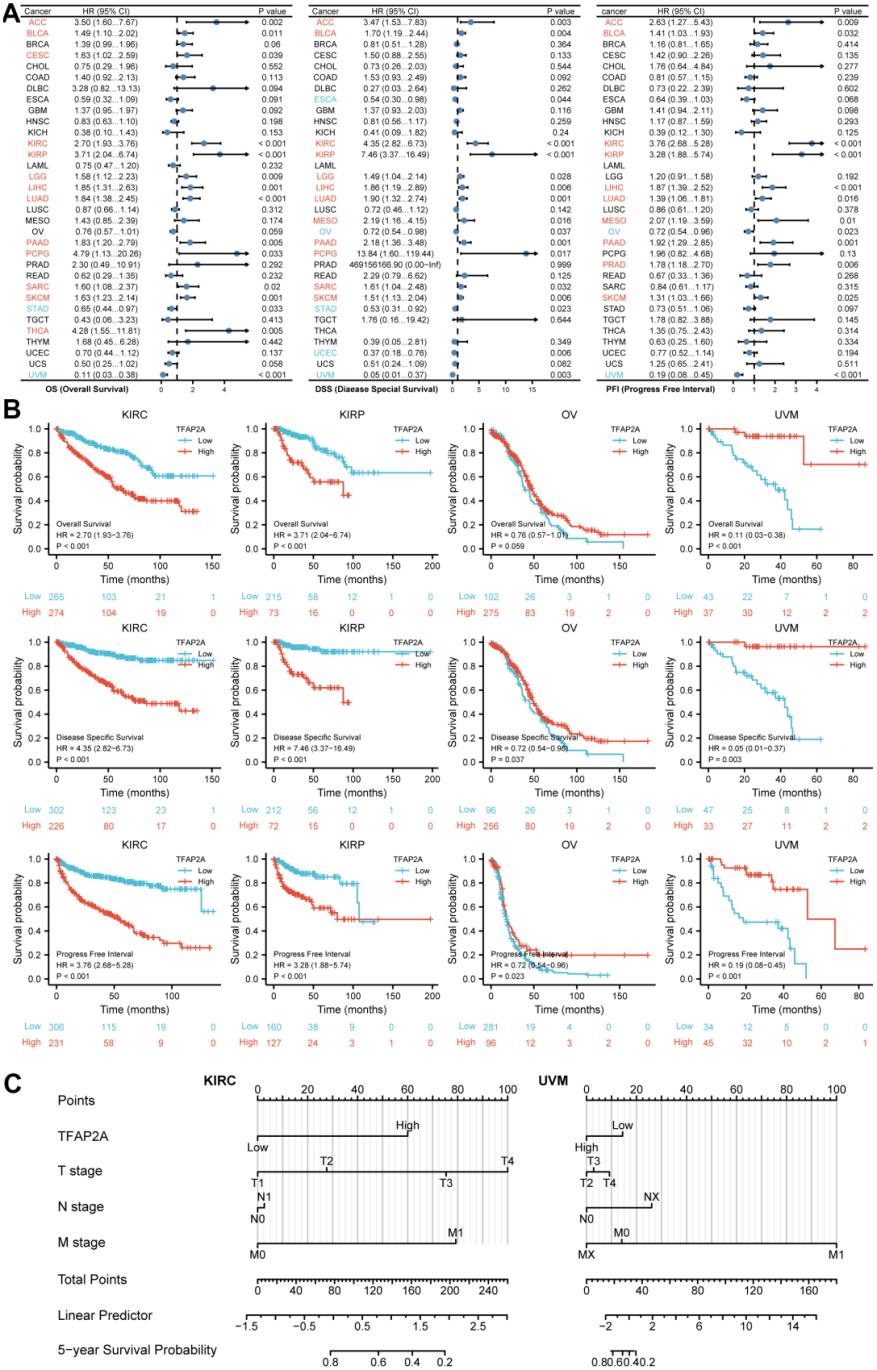
**Prognosis analysis of TFAP2A in different cancer types.** (**A**) Correlation between TFAP2A expression and OS, DSS, PFI analyzed by Cox regression respectively. HR > 1 represents high-risk cancer type and HR < 1 represents low-risk cancer type. (**B**) Kaplan-Meier survival curve of KIRC, KIRP, OV, UVM with high and low TFAP2A expression. (**C**) Nomogram predicting 5-year survival probability for KIRC and UVM.

To investigate the possible role of TFAP2A in clinical prognosis, we examined the data from the TCGA database to identify the co-relationship between TFAP2A expression with prognosis utilizing Cox regression analysis. The outcomes included OS, DSS, and PFI in many types of cancers. Results indicated that TFAP2A was positively associated with the HR in KIRC and KIRP yet negatively associated with Ovarian serous cystadenocarcinoma (OV) and UVM (Figure 2A), which would be further studied. Subsequently, higher TFAP2A expression was correlated with worse outcomes in KIRC and KIRP (HR>1). While in OV and UVM (HR<1), the reverse applied (Figure 2B). Kaplan-Meier survival curves of other human cancers were also displayed in Supplementary Figures, OS, DSS, and PFI were included (Supplementary Figure 2A–2C). Next, KIRC (HR>1) as well as KIRP (HR<1) were chosen to establish Nomogram prognostic model, respectively, which directly predicted 5-year survival probability. Consistent with previous results, higher expression of TFAP2A in KIRC was significantly associated with shorter survival whereas higher expression of TFAP2A in KIRP was significantly associated with longer survival. Ultimately, both Cox regression analysis and Nomogram prognostic model manifested that TFAP2A could be a valuable and worthy prognostic factor.

### The genetic alteration analysis of TFAP2A in various cancers

**Figure 3:**
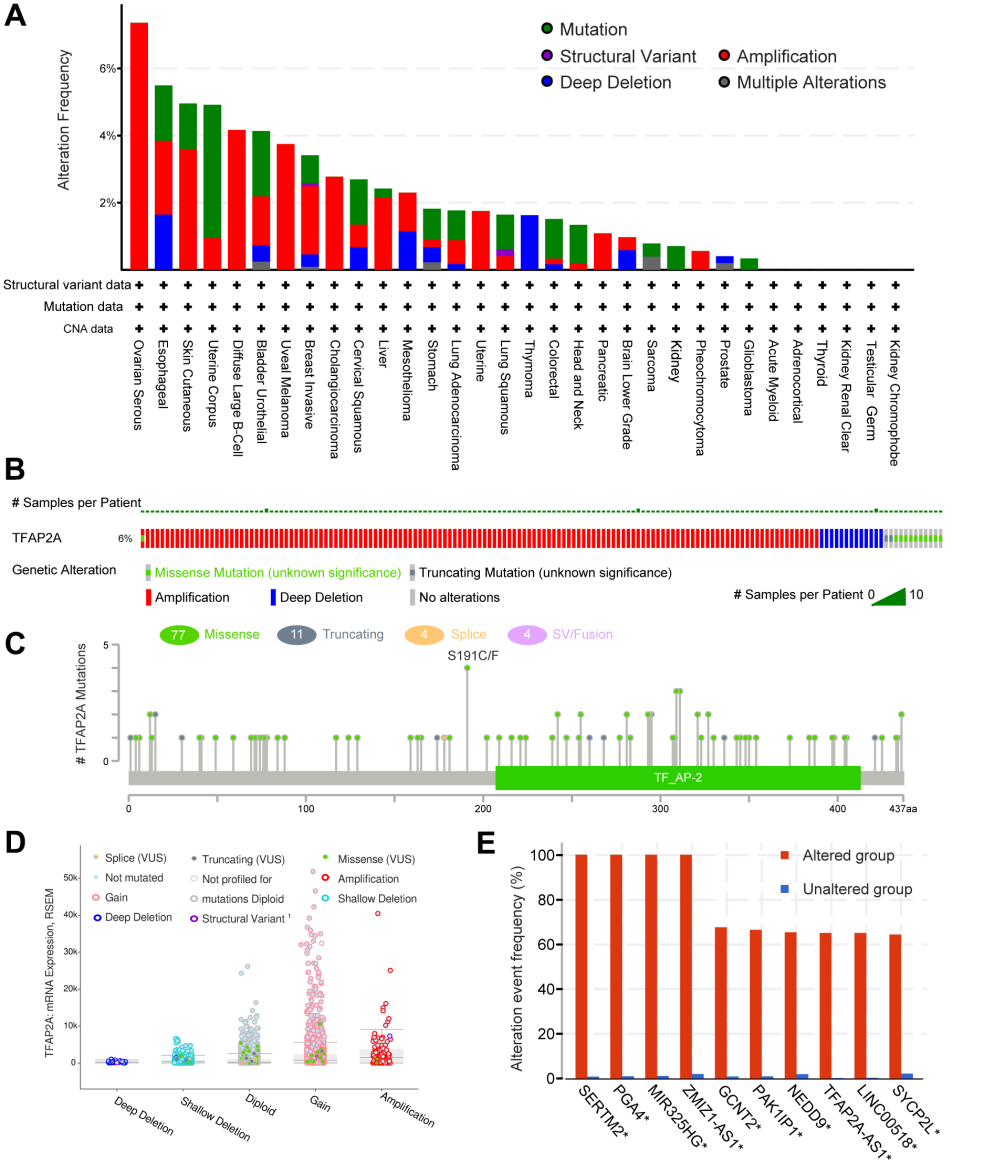
**The genomic alterations of TFAP2A.** (**A**) Details of TFAP2A genomic alterations in TCGA pan-cancer datasets; (**B**) The summary of TFAP2A genomic alterations in cancer cohort; (**C**) TFAP2A genetic mutations counts, types, and sites. (**D**) The correction of TFAP2A mRNA expression with main types of its genomic alterations; (**E**) The alteration frequency comparisons of TFAP2A related genes between TFAP2A altered and unaltered group.

The genetic alteration of TFAP2A in various cancers was then investigated by cBioPortal. The highest gene alteration frequency of TFAP2A occurred in ovarian serious carcinomas (>6%), which was all made up of amplification. In addition to ovarian serous carcinomas, more than 4% of esophageal carcinoma, skin cutaneous melanoma, uterine corpus endometrial carcinoma, diffuse large B-cell lymphoma, and bladder urothelial carcinoma obtained TFAP2A gene alteration (Figure 3A). Amplification, deep deletion, and missense mutation are the top three factors among the types of genetic alterations of TFAP2A (Figure 3B). Besides, not only were a total of 96 TFAP2A mutations that included 77 missenses, 11 truncating, 4 splices, 4 SV/fusion summarized, but also the alteration types, occurrence sites, and case numbers of TFAP2A were displayed (Figure 3C). Additionally, amplification, gain function, and diploid were TFAP2A's hypothesized copy-number modifications that were most frequently seen (Figure 3D). A higher copy number of TFAP2A was associated with increased expression. The gene alteration of SERTM2, PGA4, MIR325HG, ZMZ1-AS1, GCNT2, PAK1IP1, MEDD9, TFAP2A-AS1, LINC00518, SYCP2L was more prevalent in the altered group than unaltered group, and the alteration event frequency of SERTM2, PGA4, MIR325HG, ZMZ1-AS1 even reached 100% (Figure 3E). We also used GSEA to analyze all differentially altered genes between TFAP2A altered group and unaltered group, the top five pathways, “REACTOME DNA METHYLATION”, “REACTOME ACTIVATED PKN1 STIMULATES TRANSCRIPTION OF AR ANDROGEN RECEPTOR REGULATED GENES KLK2 AND KLK3”, “REACTOME SIRT1 NEGATIVELY REGULATES RRNA EXPRESSION”, “REACTOME PRC2 METHYLATES HISTONES AND DNA”, and “KEGG SYSTEMIC LUPUS ERYTHEMATOSUS” were displayed (Supplementary Figure 3).

### Genes co-expressed with TFAP2A and enrichment analysis

**Figure 4:**
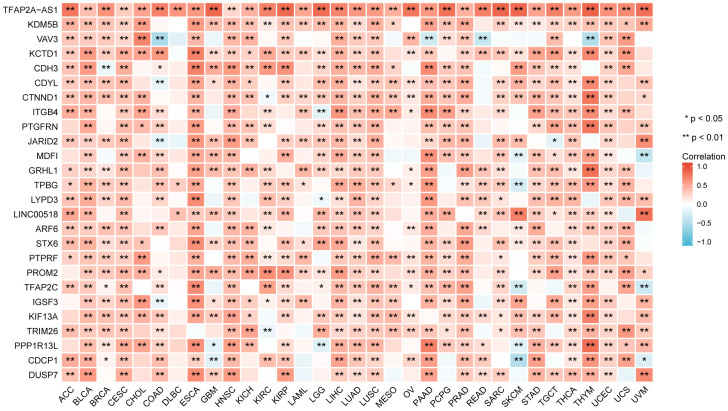
The relationship between TFAP2A expression and co-expressed genes in pan-cancer (the most relevant 26 genes).

We screened genes co-expressed with TFAP2A in 33 cancers, then estimated whether the genes co-expressed with TFAP2A in more than one half of cancer types. Following that, 26 genes were ultimately selected. We visualized the correlation of TFAP2A and its co-expressed genes by using heatmap (Figure 4). TFAP2A-AS1 undisputedly gained the highest score among all the 26 genes. KDM5B, a histone demethylase which can demethylate 'Lys-4' of histone H3, is critical for epigenetic regulation [29]. Recent research suggested that KDM5B promoted immune escape by recruiting SETDB1 to silence retroelements. Then we carried out GO term and KEGG pathway analysis (Supplementary Figure 4A). The results illustrated that the 26 genes mentioned above were primarily enriched in immune-related pathways like “Leukocyte transendothelial migration”, “Fc gamma R-mediated phagocytosis”, which had high GeneRatio. And significant histone demethylase and cell junction organization pathways.

### GSEA of differential genes between high or low TFAP2A expression groups

**Figure 5:**
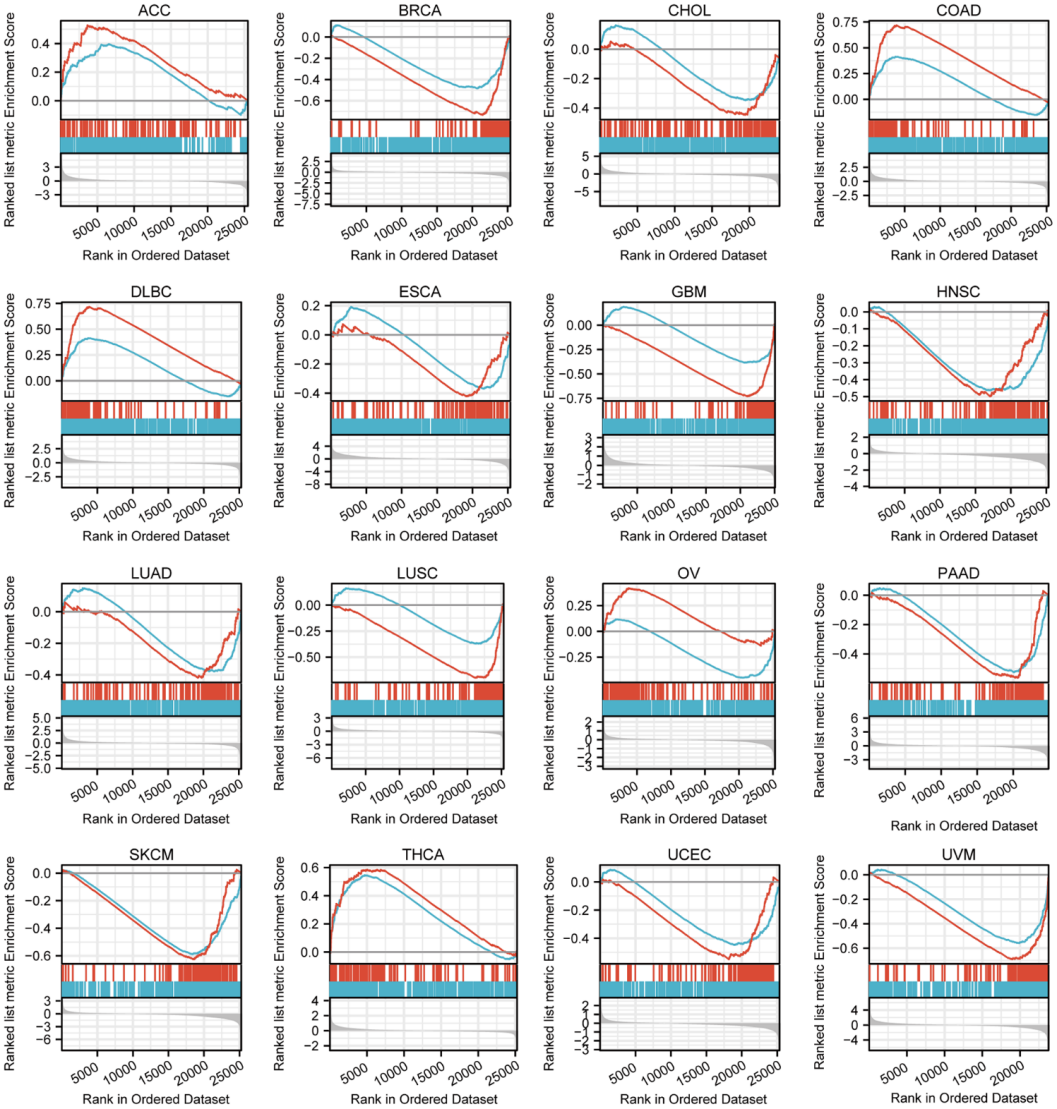
**GSEA of differential genes in high and low TFAP2A expression group.** Enrichment pathways in ACC, BRCA, CHOL, COAD, DLBC, ESCA, GBM, HNSC, LUAD, LUSC, OV, PAAD, SKCM, THCA, UCEC, UVM. The blue curve represents “REACTOME_GPCR_ LIGAND_BINDING” pathway, the red curve represents “REACTOME_IMMUNOREGULATORY_INTERACTIONS_BETWEEN_A_ LYMPHOID_AND_A_NON_LYMPHOID_CELL” pathway.

After enrichment analysis of genes co-expressed with the TFAP2A, we carried out GSEA to analyze genes that exist expression differences between low and high TFAP2A expression groups (Figure 5). Among 33 cancer types, 16 of them significantly enriched in specific pathways (p-value < 0.05 and FDR < 0.25), and we further prioritized them in each cancer type according to the absolute Normalized enrichment score (NES). Through comprehensive consideration, two of the most common and relevant pathways are “GPCR LIGAND BINDING” and “IMMUNOREGULATORY INTERACTIONS BETWEEN A LYMPHOID AND A NON-LYMPHOID CELL”. The data suggested that TFAP2A positively regulated “GPCR LIGAND BINDING” signaling pathways in Adrenocortical carcinoma (ACC), COAD, Lymphoid neoplasm diffuse large B-cell lymphoma (DLBC), OV, and THCA. TFAP2A negatively regulated “IMMUNOREGULATORY INTERACTIONS BETWEEN A LYMPHOID AND A NON-LYMPHOID CELL”, related to the control of immunological response, in BRCA, CHOL, ESCA, GBM, HNSC, LUAD, LUSC, OV, Pancreatic adenocarcinoma (PAAD), SKCM, UCEC, UVM. These results suggested TFAP2A had a complex regulation in pan-cancer.

### Correlation of TFAP2A expression with ICP genes

**Figure 6:**
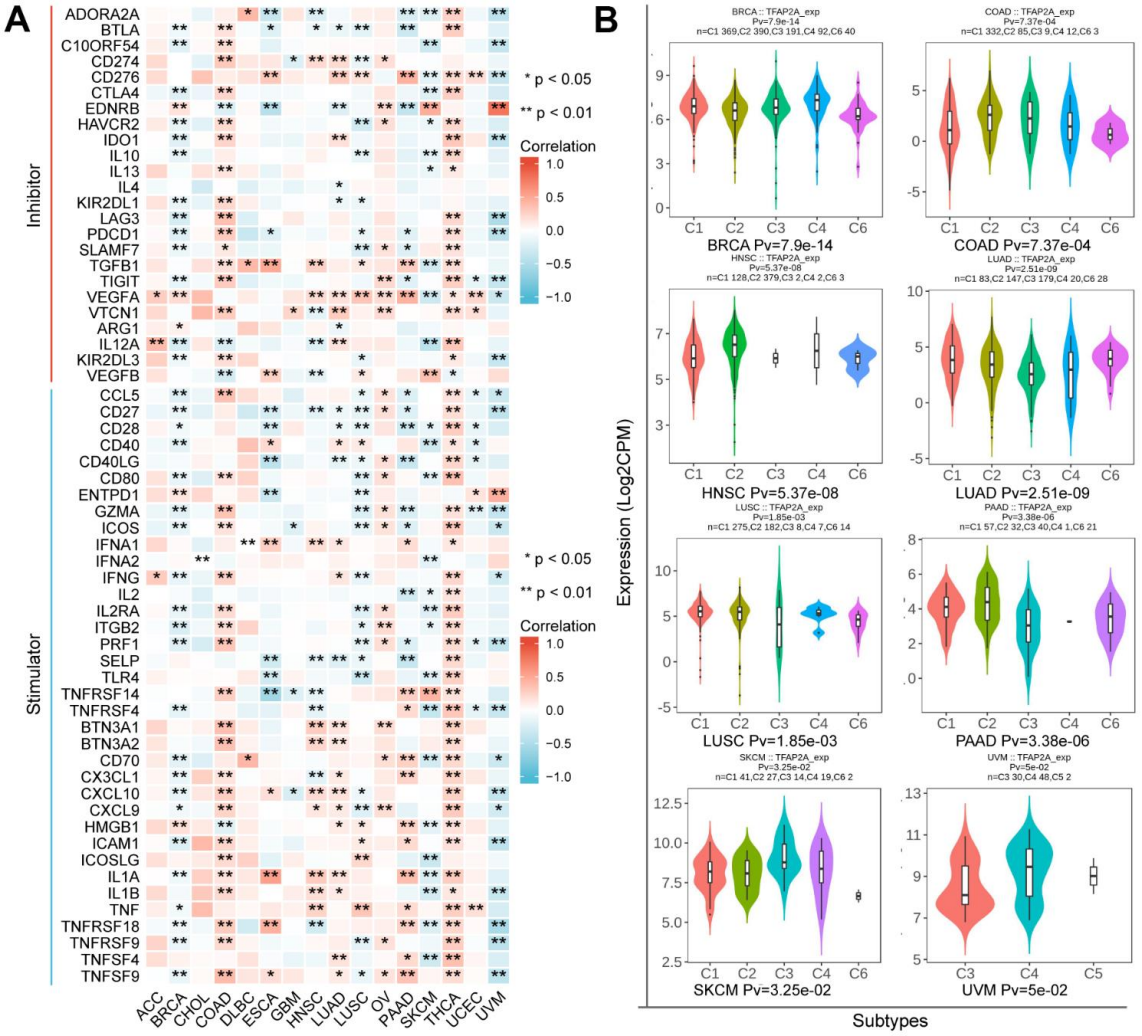
**The relationship between TFAP2A expression and immune characteristics.** (**A**) Co-expression analysis of TFAP2A with ICP genes in ACC, BRCA, CHOL, COAD, DLBC, ESCA, GBM, HNSC, LUAD, LUSC, OV, PAAD, SKCM, THCA, UCEC, UVM; (**B**) Analysis of TFAP2A expression with immune subtypes in BRCA, COAD, HNSC, LUAD, LUSC, PAAD, SKCM, UVM.

These findings prompted us to wonder whether TFAP2A contributed to tumor immunity. To figure out the link between TFAP2A expression and tumor immunity, a total of 60 ICP genes consisting of 24 inhibitors and 36 stimulators were analyzed in 16 cancer types (Figure 6A). Next, we examined the effect of TFAP2A overexpression on ICP genes’ expression. Consistent with the cancer types determined in section 7, we selected BRCA cell line MCF-7 and COAD cell line Caco2 to carry out experimental validations. Most of the ICP genes had a significant correlation with TFAP2A expression in BRCA and COAD. Eight ICP genes which have significant correlation (p<0.001) with TFAP2A expression were chosen, including CTLA4, IDO1, LAG3, PDCD1, IL12A, CCL5, PRF1, and ICAM1. Considered to be a result of low expression in cell lines, CTLA4 and PDCD1 could not be detected. Compared to the control group, all ICP genes’ expression upregulated except IL12A in MCF-7, and the levels of all ICP genes decreased except ICAM1 in Caco2 after overexpressing TFAP2A (Supplementary Figure 5), which essentially in agreement with the results of analyzation.

Immune subtypes were classified into six types, including C1 (wound healing), C2 (IFN-γ Dominant), C3 (inflammatory), C4 (lymphocyte depleted), C5(immunologically quiet), and C6 (TGF-β dominant) [25]. TFAP2A expression was closely correlated with immune subtypes in BRCA, COAD, HNSC, LUAD, LUSC, PAAD, SKCM, and UVM (Figure 6B). Considering the results, we made the assumption that TFAP2A could have an immune-related function in pan-cancer based on the aforementioned findings.

### Immune cell infiltrations and immunotherapy efficacy associated with TFAP2A level

**Figure 7:**
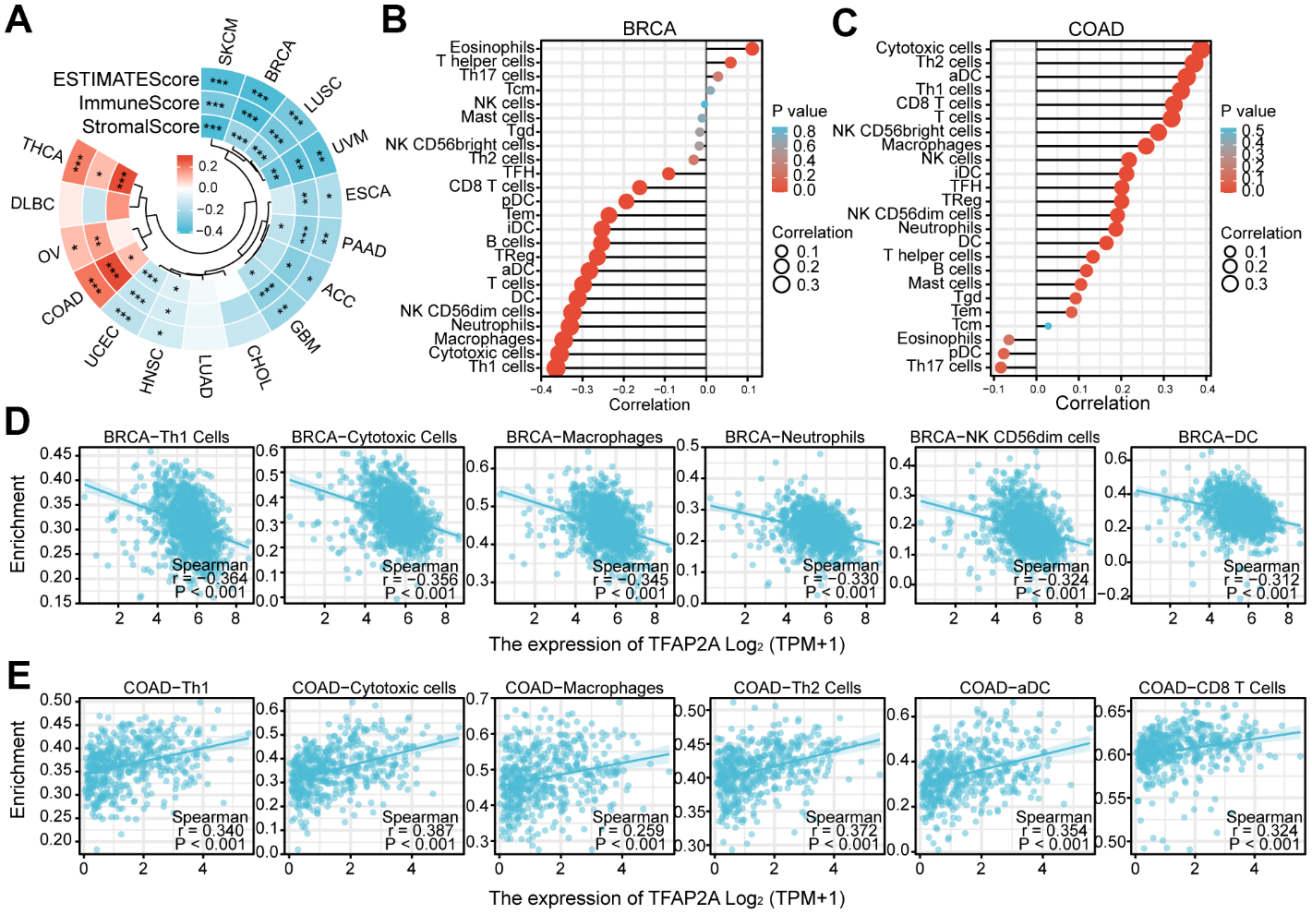
**The relationship between TFAP2A expression and infiltrating immune cells.** (**A**) The ESTIMATEScore, ImmuneScore and StromalScore in ACC, BRCA, CHOL, COAD, DLBC, ESCA, GBM, HNSC, LUAD, LUSC, OV, PAAD, SKCM, THCA, UCEC, UVM; (**B**, **C**) Lollipop chart examining the correlation of TFAP2A with immune cell infiltration in (**B**) BRCA and (**C**) COAD; (**D**, **E**) Scatter diagram illustrated the relationship between TFAP2A expression and infiltrating immune cells in (**D**) BRCA and (**E**) COAD. *p < 0.05, **p < 0.01, and ***p < 0.001.

To investigate the potential of TFAP2A in immunotherapy, we concentrated on the immunological effects of TFAP2A expression. ESTIMATE was an algorithm, which was developed by Yoshihara et al. for predicting tumor purity in the TME. The algorithm included stromal score, immune score, and estimate score. We utilized the ESTIMATE Score to evaluate TFAP2A expression in 16 cancers TME. ESTIMATEScore, ImmuneScore, and StromalScore indicated that TFAP2A expression was negatively relevant to 12 out of 16 cancers. TFAP2A expression and ESTIMATEScore showed the strongest positive correlation in COAD, whereas it exhibited the strongest negative correlation in BRCA. Thus, we chose BRCA and COAD to assess immune cell infiltration and the major infiltration of immune cells (Figure 7A). Lollipop chart and scatter diagram showed that TFAP2A expression was negatively associated with many immune cell infiltrations in BRCA, while it accelerated numerous immune cell infiltration in COAD like Th1, cytotoxic cells, macrophages, Th2 cells, aDC, and CD8 T cells (Figure 7B–7E). Immune cell infiltration in other 14 types of cancer also can be found in Supplementary Table 3.

**Figure 8:**
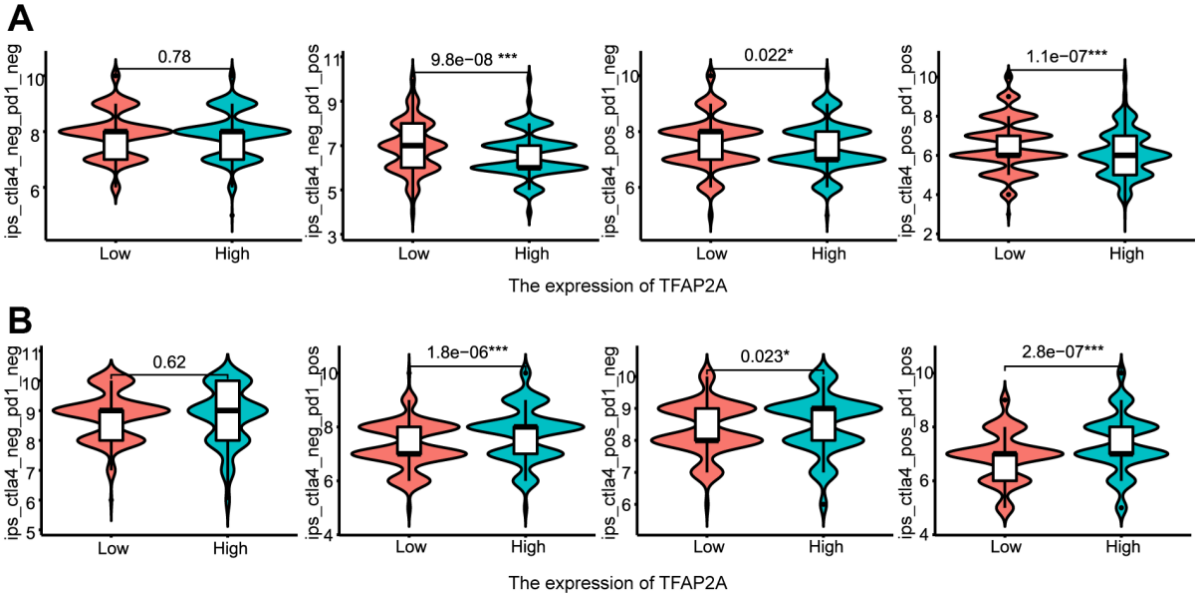
**The relationship between TFAP2A expression and immunotherapy efficiency.** (**A**, **B**) Analysis of TFAP2A expression with the efficacy of blocking CTLA4 or PD1 in (**A**) BRCA and (**B**) COAD.

Following that, we continued to take BRCA and COAD as examples (Figure 8A, 8B). Low TFAP2A expression BRCA patients were predicted to obtain better immunotherapy efficacy in receiving CTLA4 or PD-1 blockade treatment, but related to unsatisfying immunotherapy efficacy in COAD patients. Thus, on the one hand, we could detect the expression level of TFAP2A to estimate the immunotherapy response. On the other hand, TFAP2A might be used as an innovative therapeutic target to obtain a promising efficacy.

### Experimental validation of the role of TFAP2A in BRCA and COAD

4 pairs of colon tissue samples and adjacent normal colon tissue samples were collected to investigate the expression of TFAP2A and PD-L1. The protein levels of TFAP2A were elevated in three tumor tissue samples compared to the adjacent normal tissues. While one out of four tissues didn’t match the previous results. Overall, these results indicated that TFAP2A expression was positively correlated with PD-L1 expression in most of fresh colon tissues.

**Figure 9:**
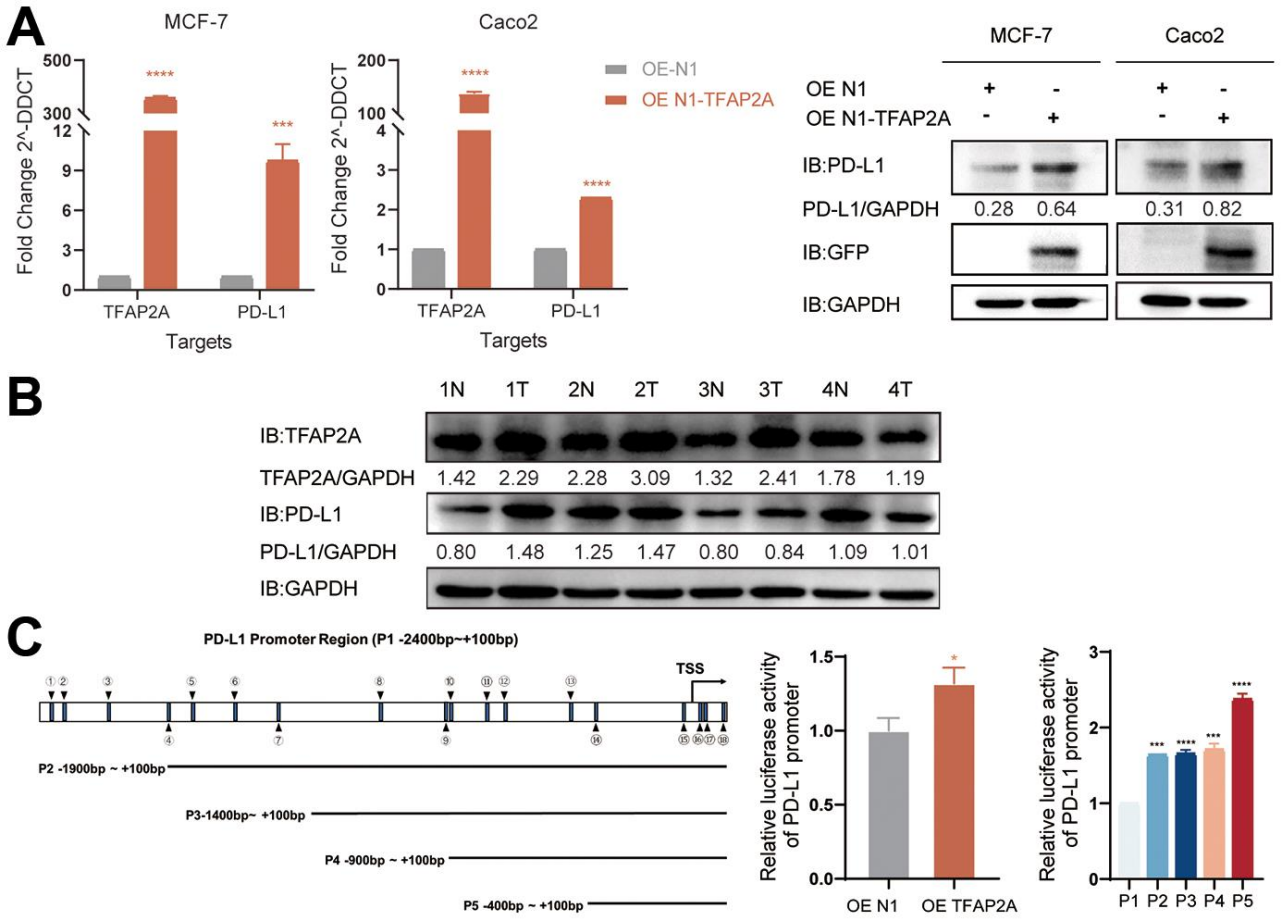
**Experimental validation of the role of TFAP2A in BRCA and COAD.** (**A**) RT-qPGR and Western blot analysis of the PD-L1 mRNA and protein levels, respectively, were determined in MCF-7 and Caco2 cells with TFAP2A overexpression; (**B**) TFAP2A protein levels in fresh colon cancer tissue (T) and adjacent normal samples (N) (4 pairs) detected by Western blot; (**C**) Schematic diagram showed the predicted binding site of TFAP2A on PD-L1 promoter and truncated PD-L1 promoter constructs. Relative luciferase activity in HEK-293FT cells co-transfected with the PD-L1 promoter and TFAP2A OE plasmid. *p < 0.05, **p < 0.01, ***p < 0.001, and ****p < 0.0001.

Considering TFAP2A’s crucial function and its tight relationship with significant PD-1 blockade treatment efficacy disparities in BRCA and COAD patients. We interfered with the expression of TFAP2A to validate the impacts of TFAP2A expression on PD-L1 expression, MCF-7 and Caco2 cell lines were chosen to carry out cell experiments. The TFAP2A overexpression promoted the expression of PD-L1 on mRNA and protein levels in both MCF-7 and Caco2 cell lines (Figure 9B). Based on the above experimental results that TFAP2A and PD-L1 might have a regulatory relationship. Considering the basic function of TFAP2A, a transcriptional factor, we speculated a possibility that TFAP2A directly binds to the PD-L1 promoter to promote its transcription. The results of the dual-luciferase reporter assay indicated that the luciferase activity driven by the PD-L1 promoter was dramatically enhanced by TFAP2A OE. Next, we explored the JASPAR database and found there are 18 TFAP2A-binding regions on PD-L1 promoter (P1 -2400 to +100bp) with a relative profile score threshold of 80%. We constructed a series of truncated reporter plasmid P2, P3, P4, and P5 according to the full-length promoter of PD-L1 (P1) to identify a specific binding site. The P5, which ranges from -400 to +100bp, had the strongest ability to drive the luciferase activity. Our results suggested that TFAP2A acts as a transcriptional activator in the PD-L1 expression regulation and the essential binding site for the this may be in the -400 to +100bp region of PD-L1 promoter.

## DISCUSSION

The TFAP2A gene encodes TFAP2α protein and is localized predominantly in the nucleus. As its name suggests, TFAP2α works mainly via recognizing the consensus DNA sequence 5’-GCCNNNGGC-3’ of target genes and regulating their transcription [1]. Numerous studies have shown that TFAP2α influenced tumor progression by interacting with diverse molecules and regulating different pathways in multiple cancers. TFAP2α can also work by interacting with other types of molecules such as miRNAs, proteins, or long non-coding RNAs. MiR-200b, which is negatively correlated with the expression of TGF-β and is able to bind the 3′-UTR of TFAP2α in CHOL. Concurrently, TFAP2α binds to the promoter of TGFB1 and promotes its transcription, which enhances EMT [30]. As we mentioned above, Wwox interacts with TFAP2α proteins. Wwox is a WW domain-containing protein and acts as a tumor suppressor. Wwox binds the proline-rich motif of TFAP2α and brings it out of the nucleus to reduce its transcriptional activity and subsequently affect tumor progression [31]. Besides, TFAP2α can bind to a 513-nt region at the 3’ end of long noncoding RNA CCAL, which promotes ubiquitin-dependent TFAP2α degradation by the proteasome and activates the cancer-promoting Wnt/β-catenin pathway in Colorectal cancer (CRC) [32].

The function of TFAP2A in prognosis and tumor immunity in pan-cancer was still controversial and obscure. TFAP2A has been found to act as a transcription factor regulating the level and activity of multiple downstream signaling proteins. It exerts either transcriptional activation or repression function. For example, TFAP2A has been found to directly bind to KRT16 and ITPKA promoter, resulting in hyper-expression of them in LUAD [33, 34]. Additionally, TFAP2A prevented the deacetylation activity of the NuRD Complex on E2F pathway promoters by competing with the same chromatin fragments, which drove hyper-acetylation of promoter nucleosomes and facilitated transcription in melanoma [35]. Which impact ultimately predominates will depend on other factors or on TFAP2A expression levels because TFAP2A may activate some of its targets while repressing others [36]. Overall, the functional mechanism of TFAP2A in cancer appears to be complex and context-dependent, with oncogenic and tumor-suppressive functions depending on the specific cancer type, interaction molecular and cellular context. In this study, we focused our study on the involvement of TFAP2A in pan-cancer to identify its common functional roles. We first determined the expression of TFAP2A in normal and tumor tissues. TFAP2A expression is significantly higher in most cancer types except ACC, PRAD, Sarcoma (SARC), Testicular germ cell tumors (TGCT), and THCA, which is consistent with the analysis from the TIMER2 database and those in previous studies, such as in CHOL, Esophageal cancer (EC), Gastric cancer (GC), HNSC, Non-small cell lung cancers (NSCLC), OC, etc. [32, 37–41], suggests TFAP2A indeed promote oncogenesis and tumor progression. As we know, proteoforms are molecular actuators of gene function, so we explored the HPA database to obtain Immunohistochemistry (IHC) images. The IHC images showed that the protein expression levels of TFAP2A in several cancer types were higher, which was in accordance with the mRNA levels from other databases. It is almost consistent with the prediction of bioinformatic results that the levels of TFAP2A and PD-L1 protein were upregulated in three out of four colonic cancer tissues. PD-L1 expression was also positively related to TFAP2A expression. More colonic tissues and other kinds of cancer tissues are needed to further confirm their expression correlations. TFAP2A, especially in combination with PD-L1, may be employed as a predictive biomarker for prognosis in patients with COAD. The connection between TFAP2A expression and patient prognosis was then shown. In roughly half of cancer types, TFAP2A expression was strongly linked to a poorer prognosis. Among them, high TFAP2A expression meant significantly worse prognosis with a few exceptions, which was supported by other studies and proved that TFAP2A perhaps could be a useful pan-cancer prognosis biomarker [14, 42–44]. It was interesting to note that TFAP2A mRNA expression in cognate cancer tissues was higher, whereas better outcomes in patients bearing high TFAP2A expression in some cancer types. The phenomenon can be interpreted as tissue and cancer specificity of cancer-related genes [45].

Genome instability and mutation are well-known to be important causes of tumor development and progression [46]. TFAP2A gene is located on chromosome 6p24.3 [1]. Several studies have shown TFAP2A gene alternation may cause diseases. In a study about patients with nonsyndromic cleft lip and palate, Single-nucleotide variants (SNVs) were identified on TFAP2A in the non-coding region [47]. Another study identified a novel susceptibility allele, which lies on 6p24 and within a region containing TFAP2A, was related to risk in BRCA2 mutant carriers rather than general population or BRCA1 mutant carriers [48]. To understand TFAP2A genetic alterations in pan-cancer, we explored the cBioPortal database. The most altered type of TFAP2A was amplification, which may be one of the causes of the increase of TFAP2A mRNA expression in cancer. Besides, it was found that genes altered by co-occurrence with TFAP2A including TFAP2A-AS1, GCNT2, PAK1IP1, TMEM14C, C6ORF52, etc. We further performed the analysis with GSEA of these genes and found them closely related to DNA and histone methylation in cancer.

In order to get further understanding of the common functions of TFAP2A in pan-cancer, we performed enrichment analysis of co-expressed and differentially expressed genes with TFAP2A in pan-cancer and collated the data. Not surprisingly, the gene most relevant to TFAP2A was TFAP2A-AS1, a long non-coding RNA (lncRNA) that has been reported to promote Oral squamous cell carcinoma cell growth and migration by regulating the expression of TFAP2A [49]. The second gene most related to TFAP2A was KDM5B, a histone demethylase which has been proven essential for immune evasion in melanoma [29]. The result of GO term and KEGG pathway enrichment analysis showed cell adhesion pathway significantly correlated with TFAP2A, as well as histone demethylation and immune-related genes co-expressed with TFAP2A. GSEA likewise outputs enrichment of immunoregulation and GPCR ligands pathways. The negative relationship between TFAP2A in most cancer types indicated that TFAP2A may play a crucial role in tumor immune escape. It was noteworthy that TFAP2A might be involved in epigenetic modifications regulating, such as mentioned DNA and histone methylation, which were imbalanced in many cancers and could be considered for further investigation [50]. Here, it’s worth mentioning that many studies have shown that GPCRs are aberrant expression and activation in cancer, which is connected to inflammation and immune cells evasion in cancer [51, 52]. A study about immune-related gene identification in COAD determined 11 prognosis-related genes. The result showed immune-related TDGF1 was positively modulated by TFAP2A and reminded us of the function of TFAP2A in tumor immunity [53].

These results strongly suggested the potential role of TFAP2A in tumor immunity. Following the above, we explored in more depth the relationship between TFAP2A and tumor immunity in 16 cancer types. First, co-expression analysis showed a significant correlation between TFAP2A and ICP genes, which suggested that TFAP2A might coordinate ICP gene activity in different signaling pathways [54]. RT-qPCR was conducted to further clarify the relationship between TFAP2A and ICP genes. Although the experimental data is a slight discrepancy from the predicted results, 5 out of 6 are the same tendency as them. The regulatory mechanism between TFAP2A and ICP genes expression is complex, which calls for in-depth exploration. The result prompted the potential target role of TFAP2A in immunotherapy. Then, we found TFAP2A expression related to immune subtypes, C1-C6, in one-half of 16 cancer types. C1-C3 subtypes were sensitive to immunotherapy, and patients might not benefit from immune neoadjuvant therapy in the latter three subtypes [25]. Therefore, TFAP2A has the potential to be a treatment guidance for evaluating immune neoadjuvant therapy effects. A recent study about pancreatic cancer showed that solasonine is directly bound to TFAP2A and suppressed its protein levels [55]. This makes it possible to combine immune checkpoint inhibitors or agonists of stimulatory ICP with drug-targeted TFAP2A [56, 57]. Next, we discovered a connection between TFAP2A and the immune, stromal, and ESTIMATE scores of the TME. Among them, we chose the cancer types which were the strongest negative and positive correlated with the immune score, BRCA, and COAD, to evaluate the infiltration of Tumor-infiltrating lymphocytes (TILs). The commonality between both cancer types was that they were significantly associated with Th1 and Cytotoxic Cells infiltration, which usually predicted better clinical outcomes [58]. A study showed that the regulator of G protein signaling (RGS)1 was upregulated in helper Th1 cells and Cytotoxic T lymphocytes (CTLs), which reduced their trafficking to and survival in breast tumors [59]. The study reminded us of the potential regulatory role of TFAP2A in Th1-related and GPCR ligand pathways. To validate the value of TFAP2A in immunotherapy efficacy, we assessed the differences in the predicted effect of immunotherapy between low and high TFAP2A expression in BRCA and COAD patients. Anti-PD-1/PD-L1 and anti-CTLA4 have now been universally acknowledged as significant breakthroughs in tumor therapy [60]. The discrepancy of predicted effect in two groups patients was most significant when receiving PD-1 blockade treatment. For therapeutic purposes, defining the potential function of TFAP2A in immunotherapy, as well as if it’s possible to employ TFAP2A as an effective biomarker guiding clinical treatment, are still expected. Given the big differences in the predicted efficacy of receiving PD-1 blockade treatment or not, the obvious correlation of TFAP2A and PD-1 was shown. A tremendous number of researches indicated that high levels of PD-L1 expression was seen in a few cancers and exploited the PD-L1 and/or PD-1 signaling alternation to induce T-cell-mediated immune escape [61]. To further clarify whether TFAP2A expression had influences on tumor immunity, we studied the effect of TFAP2A interference on PD-L1 expression using two cancer cell lines. RT-qPCR and WB were proceeded in MCF-7 and Caco2. Our results showed that higher TFAP2A expression facilitated the mRNA and protein expression of PD-L1. The binding sites where TFAP2A acts as a facilitator are mainly located in the PD-L1 promoter region ranging from -400 to +100bp through luciferase reporter assay. Our observations strongly suggested that TFAP2A is a transcription factor for PD-L1. The hypothesis requires further experimental validation by luciferase reporter assay, chromatin immunoprecipitation, etc. Besides, TFAP2A and PD-L1 positively correlated with each other at transcriptional level. And we guessed one of the reasons was there might be also a transcription factor for TFAP2A which activated its transcription. It’s reported that PD-L1 protein expression can be affected by various kinds of factors, including some post-translational modifications like ubiquitination, glycosylation, phosphorylation, and palmitoylation [62], which perhaps can explain more than one PD-L1 bands detected in each lane. The smaller molecular weight bands corresponded to the unmodified form and were consistent with the results of its mRNA expression. The large molecular weight bands corresponded to the modified form and the variation in protein level might be responsible for protein stability. We leave these questions to future study about how TFAP2A interacts with PD-L1 and whether TFAP2A is responsible for the modification and effect the protein stability of PD-L1.

Even though we conducted a comprehensive and progressive examination of TFAP2A and employed different databases for cross-validation, there are still several limitations in our work. First, to increase confidence in our findings, *in vivo* and more *in vitro* experiments are required to validate our hypothesis about the probable function of TFAP2A. Next, the exact mechanism is still unknown although we have concluded that TFAP2A influences TME and immunotherapy efficacy. Finally, we did not analyze and elucidate the potential role of TFAP2A in cancer-associated biological processes other than immune regulation, such as DNA and histone methylation.

## Supplementary Material

Supplementary Figures

Supplementary Tables
