# Supplementary material for: Potential prognosis and immunotherapy predictor TFAP2A in pan-cancer
Source: Aging (Albany NY). 2024 Jan 23;16(2):1021–48. doi: 10.18632/aging.205225 (PMC10866441; doi:10.18632/aging.205225)
Supplement: Supplementary Figures [file aging-16-205225-s001.pdf]

SUPPLEMENTARY FIGURES

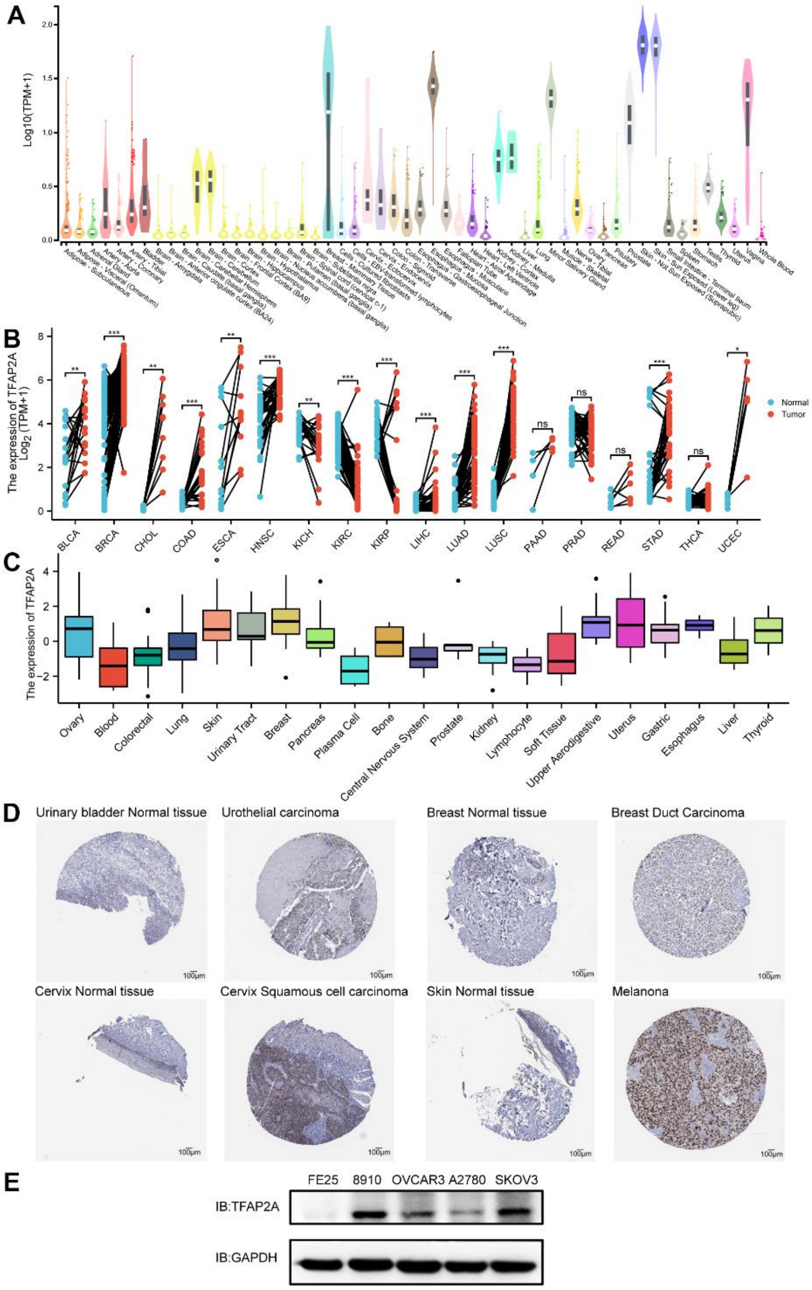

Supplementary Figure 1. Different expression of TFAP2A. (**A**) TFAP2A mRNA expression in normal tissue from GTEx database; (**B**) Differential TFAP2A mRNA expression between paired TCGA cancers and normal tissues. The red column represents cancer samples and the blue column represents normal samples. Normal group was normal tissue in TCGA and GTEX database; (**C**)The TFAP2A protein expression in different cancer cell lines from CCLE database. (**D**) Immunohistochemistry of TFAP2A in BLCA, BRCA, CESC, SKCM and corresponding normal tissues from the HPA database (antibody: CAB000326): in urinary bladder (staining: not detected; intensity: negative; quantity: none)and in BLCA tissues (staining: medium; intensity: strong; quantity: <25%); In breast (staining: low; intensity: moderate; quantity: <25%) and in BRCA tissues (staining: high; intensity: strong; quantity: >75%); In cervix (staining: medium; intensity: moderate; quantity: 75%-25%) and in CESC tissues (staining: medium; intensity: strong; quantity:<25%); in skin (staining: medium; intensity: moderate; quantity: >75%) and in SKCM tissues (staining: high; intensity: strong; quantity: >75%); (**E**) Western blot analysis of the constitutive expression of TFAP2A in normal ovarian epithelial cell line FE25 and ovarian cancer cell lines. *p < 0.05, **p < 0.01, and ***p < 0.001.

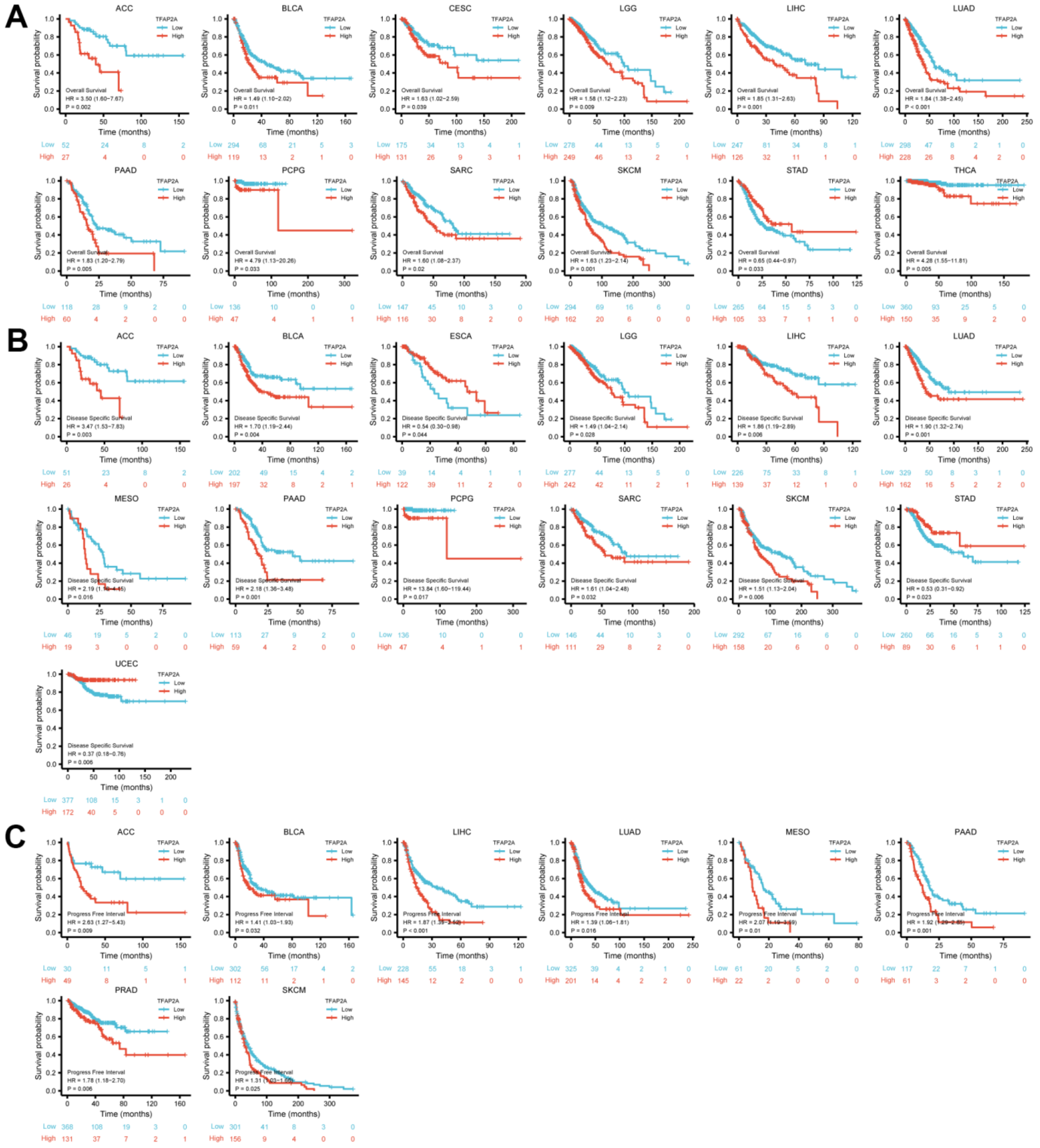

Supplementary Figure 2. Kaplan-Meier survival curve of human cancers with high and low TFAP2A expression. (**A**) OS of ACC, BLCA, CESC, LGG, LIHC, LUAD, PAAD, PCPG, SARC, SKCM, STAD, THCA with high and low TFAP2A expression; (**B**) DSS of ACC, BLCA, ESCA, LGG, LIHC, LUAD, MESO, PAAD, PCPG, SARC, SKCM, STAD, UCEC with high and low TFAP2A expression; (**C**) PFI of ACC, BLCA, LIHC, LUAD, MESO, PAAD, PRAD, SKCM with high and low TFAP2A expression.

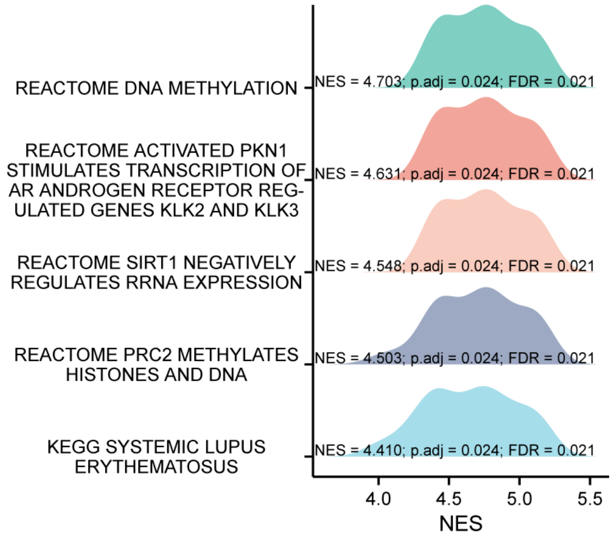

Supplementary Figure 3. GSEA of differentially mutated genes between TFAP2A altered group and unaltered group.

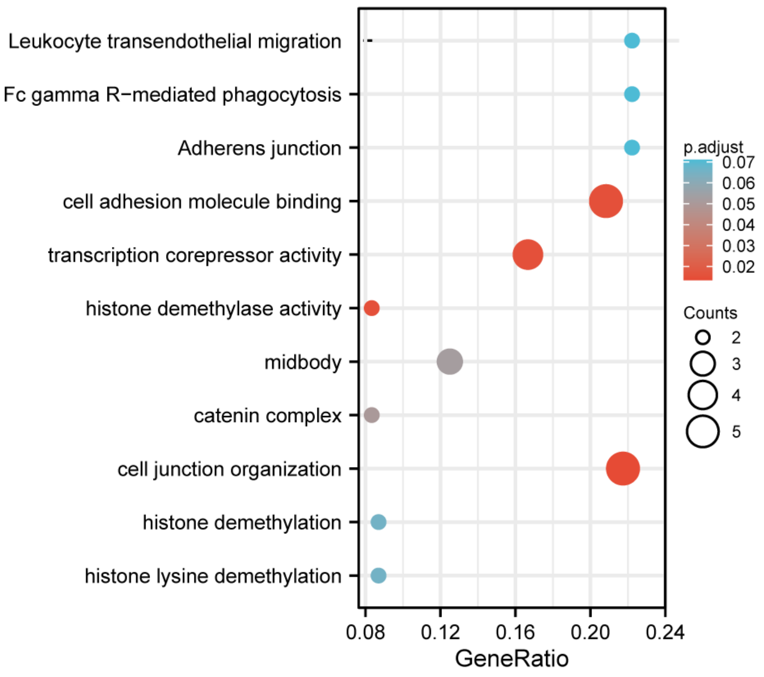

Supplementary Figure 4. GO term and KEGG pathway enrichment analysis of co-expressed genes with TFAP2A.

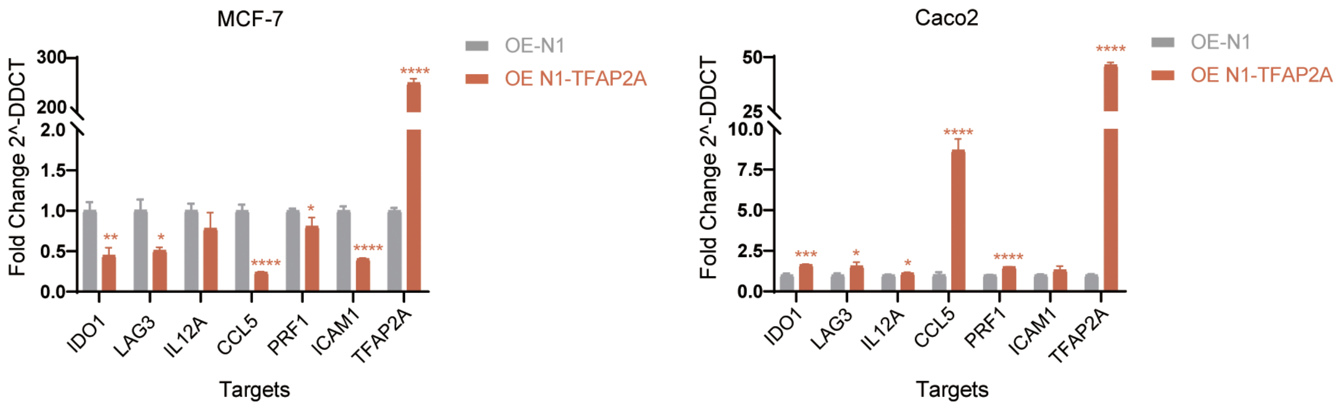

Supplementary Figure 5. Detection of six ICP genes' levels after TFAP2A overexpression in MCF-7 and Caco2 by RT-qPCR; *p < 0.05, **p < 0.01, and ***p < 0.001.
