# Supplementary material for: Potential prognosis and immunotherapy predictor TFAP2A in pan-cancer
Source: Aging (Albany NY). 2024 Jan 23;16(2):1021–48. doi: 10.18632/aging.205225 (PMC10866441; doi:10.18632/aging.205225)
Supplement: Supplementary Tables [file aging-16-205225-s002.pdf]

## SUPPLEMENTARY TABLES

**Supplementary Table 1. The correlation between TFAP2A expression and infiltrating immune cells in ACC, CHOL, DLBC, ESCA, GBM, HNSC, LUAD, LUSC, OV, PAAD, SKCM, THCA, UCEC, UVM.**

| | ACC | CHOL | DLBC | ESCA | GBM | HNSC | LUAD | LUSC | OV | PAAD | SKCM | THCA | UCEC | UVM |
|---|---|---|---|---|---|---|---|---|---|---|---|---|---|---|
| aDC | -0.0119 | 0.0311 | 0.076 | -0.0429 | -0.2658*** | 0.1248** | 0.1132** | -0.1847*** | 0.1571** | -0.0249 | -0.2668*** | 0.1249** | 0.0103 | -0.3809*** |
| B cells | -0.1997 | -0.1109 | -0.1792 | -0.3292*** | -0.0836 | -0.1491*** | -0.1241** | -0.3088*** | 0.1075* | -0.2144** | -0.1579*** | 0.1317** | -0.0686 | -0.2074 |
| CD8 T cells | -0.3361** | -0.2129 | -0.2765 | -0.2376** | 0.0499 | -0.0894* | -0.125** | -0.2204*** | 0.1387** | -0.2137** | -0.096* | 0.1965*** | -0.0891* | -0.002 |
| Cytotoxic cells | -0.2947** | -0.2311 | -0.1711 | -0.0456 | -0.2981*** | -0.1564*** | -0.0685 | -0.3204*** | 0.1611** | -0.291*** | -0.2006*** | 0.0771 | -0.2009*** | -0.3821*** |
| DC | -0.2205 | -0.1367 | 0.0129* | -0.1264 | -0.2132** | -0.16*** | -0.172*** | -0.2476*** | 0.1614** | -0.195** | -0.1626*** | 0.1437** | -0.1017* | -0.1835 |
| Eosinophils | -0.157 | -0.512** | -0.2054 | -0.5642*** | -0.1447 | 0.0128 | -0.1992*** | -0.3001*** | 0.0172 | -0.3718*** | -0.1179* | 0.3074*** | -0.0648 | -0.2331* |
| iDC | -0.2167 | -0.0093 | -0.1399 | -0.0392 | -0.187* | -0.1435** | -0.1737*** | -0.3792*** | 0.0715 | -0.2377** | -0.3453*** | 0.185*** | -0.0881* | -0.2789* |
| Macrophages | -0.227* | -0.0193 | -0.0486* | -0.0386 | -0.2506** | -0.119** | -0.0855* | -0.3176*** | 0.0575 | -0.0766 | -0.3864*** | 0.1079* | -0.0583 | -0.4255*** |
| Mast cells | -0.1284 | -0.0955 | 0.045* | -0.2941*** | -0.0718 | -0.1265** | -0.2024*** | -0.3157*** | 0.0277 | -0.3485*** | -0.2279*** | 0.1346** | -0.0255 | -0.3164** |
| Neutrophils | -0.1552 | 0.0414 | 0.0062** | -0.2031** | -0.1833* | 0.093* | -0.0236 | -0.1975*** | 0.146** | -0.1496* | -0.2375*** | 0.0678 | 0.0408 | -0.3409** |
| NK CD56bright cells | -0.3455** | 0.0324 | -0.0601 | -0.3471*** | -0.1915* | -0.2606*** | 0.0275 | 0.0136 | 0.053 | 0.2771*** | -0.1525*** | 0.0753 | -0.0977* | -0.2395* |
| NK CD56dim cells | -0.092 | -0.1115 | 0.1701 | 0.25** | -0.2563*** | 0.0227 | 0.1073* | -0.1439** | 0.1732*** | -0.0856 | -0.3123*** | 0.0948* | -0.1049* | -0.4691*** |
| NK cells | -0.0647 | 0.4293** | 0.0084** | 0.1271 | -0.2709*** | -0.1462** | 0.079 | 0.0708 | -0.0414 | -0.1085 | -0.1512** | 0.0165 | 0.009 | -0.204 |
| pDC | -0.2412* | -0.3959* | -0.0856 | -0.514*** | 0.0094 | -0.3327*** | -0.1099* | -0.1987*** | 0.0461 | -0.3113*** | -0.3251*** | 0.0817 | -0.122** | -0.2523* |
| T cells | -0.2983** | -0.1876 | -0.1779 | -0.2887*** | -0.2323** | -0.1025* | -0.0766 | -0.3223*** | 0.1656** | -0.322*** | -0.2327*** | 0.1255** | -0.1476*** | -0.4195*** |
| T helper cells | 0.025 | -0.2633 | -0.0424* | -0.16* | -0.1086 | 0.1407** | -0.0194 | -0.1176** | 0.1232* | -0.0956 | -0.2449*** | 0.2024*** | -0.0034 | -0.1389 |
| Tcm | 0.0653 | -0.2232 | -0.1659 | 0.402*** | 0.1082 | 0.2179*** | -0.0571 | 0.0592 | 0.1172* | -0.084 | -0.1351** | 0.1837*** | 0.1377** | 0.0207 |
| Tem | -0.0886 | -0.1104 | -0.1344 | -0.5188*** | 0.0941 | -0.1586*** | -0.1342** | -0.1038* | 0.1313* | -0.1389 | -0.0322 | 0.0322 | -0.0873* | -0.1192 |
| TFH | -0.211 | -0.0165 | -0.2302 | -0.174* | 0.0984 | -0.0307 | -0.1301** | -0.2556*** | 0.0294 | -0.349*** | -0.3006*** | 0.2019*** | -0.0548 | -0.3773*** |
| Tgd | -0.0889 | -0.0082 | 0.0038** | 0.0793 | 0.0311 | 0.1851*** | 0.0317 | -0.2336*** | -0.1038* | -0.1999** | -0.2453*** | 0.1336** | -0.0131 | -0.5756*** |
| Th1 cells | -0.11 | -0.0206 | 0.0319* | -0.0735 | -0.0521 | 0.1011* | -0.0253 | -0.2637*** | 0.1208* | -0.0619 | -0.341*** | 0.2395*** | -0.1164** | -0.5636*** |
| Th17 cells | -0.0772 | -0.2342 | -0.0299* | -0.5629*** | -0.1172 | 0.0138 | -0.0981* | -0.1642*** | 0.1109* | -0.2741*** | -0.1342** | 0.0729 | -0.0152 | 0.3918*** |
| Th2 cells | 0.3564** | 0.0625 | -0.0725 | 0.1119 | 0.118 | 0.1258** | 0.2262*** | -0.1154** | 0.0834 | 0.2516*** | -0.1307** | 0.1733*** | 0.1428*** | -0.5833*** |

(Spearman correlation coefficient and P-Value, *p < 0.05, **p < 0.01, and ***p < 0.001.)

**Supplementary Table 2. Sequence of primers.**

| Primer name | Sequence |
|---|---|
| TFAP2A-F | GACCTCTCGATCCACTCCTTAC |
| TFAP2A-R | GAGACGGCATTGCTGTTGGACT |
| PD-L1-F | TGCCGACTACAAGCGAATTACTG |
| PD-L1-R | CTGCTTGTCCAGATGACTTCGG |
| GAPDH-F | CGGAGTCAACGGATTTGGTCGTAT |
| GAPDH-R | AGCCTTCTCCATGGTGGTGAAGAC |
| IDO1-F | GCCTGATCTCATAGAGTCTGGC |
| IDO1-R | TGCATCCCAGAACTAGACGTGC |
| LAG3-F | GCAGTGTACTTCACAGAGCTGTC |
| LAG3-R | AAGCCAAAGGCTCCAGTCACCA |
| IL12A-F | TGCCTTCACCACTCCCAAAACC |
| IL12A-R | CAATCTCTTCAGAAGTGCAAGGG |
| CCL5-F | ACACACTTGGCGGTTCTTTCGG |
| CCL5-R | CCTGCTGCTTTGCCTACATTGC |
| PRF1-F | ACTCACAGGCAGCCAACTTTGC |
| PRF1-R | CTCTTGAAGTCAGGGTGCAGCG |
| ICAM1-F | AGCGGCTGACGTGTGCAGTAAT |
| ICAM1-R | TCTGAGACCTCTGGCTTCGTCA |

**Supplementary Table 3. The sequences of five PD-L1 promoter region and predicted binding sites.**

| Site | Sequences |
|---|---|
| P1 (−2400 to +100 bp) | ATGACTCACCTGAGGACAATGTTAAAATGTTGACCTCCAAGACCCTGCCCCCAAGTTTGGATCCCAGGCAGAACCCTGGA<br>ATCTGTATTTTAAACATTAACCCCAAGTCGAGTGTAATGCAAGTAACCATCAGAGACACTCTGAGAAACAGCACATTGGCA<br>ATGATGAATTTATATGTCTAAAAATGAATAGAGTAGATGTTACATGAATAGGAAGTGGTGGTATTCAAGATGACCATAGTATC<br>TAGCTCTTCAGCCCTGGCTCCCCACTGCTCTTCTCCCATCTCAGCACTTCTCCATCTATTTCGTCCAAATAAAAAAATTCACA<br>CAGAGTTTCAGGACTTAACCCCCCACTCATTAACCATCTGTTTTGCTTTACATATTTTTCTGAGGTAATAAAATTTCTCTTTTT<br>CTAAACACAGCCTGTTTTTCAATCTCCGGGTAGTTGATCAATTGTATGGGAAAATGAATGGCTGAAGGGTAGAAACAGGTG<br>GGAAAGATGAACAAAAACACGAATCCTCACATTACTAATACGCAAATCACTGAGCAGCAAGCTGAGCAAATACCCTCAAT<br>TCCCATCAGCAACTTTAGAGAAAGGCAAATTCCGTTTGCCTCATTGATCATTTAGGTAGACCCTGAACACTGCTTTCATAAA<br>ACAAAAACAAAATACCCATCCCCAGTTTAAAAAATTATTCATAGATCATCCAGGCCATCTAGGAGGATATGATTTAATCCTG<br>GCTACTTGGTAAATTATTTGCCCAAGTTAACTCAGCTAGTTAGTGGTAGATGGCTCTGAAGCCAGTTGTTTTTTTTTGTTTTG<br>TTTTTTGCAGACCTCAAGAGTCATGATGAACTAGCAGATCATAAAGTTTATGCCCTGGGTCTTGACCATTTTTAGAAAAATA<br>AAACATTAAATGAAAATATCAGAGGGCATTGCAGATAGTAGATCTAAGTATTTTTTCATGAAACTTGTTGTACATGTGTGTGT<br>CATACACAGACTATATATATGCAGTACCTGTAAACTGTATTGCCACATAATGTCTATATTTTCCTAGAGGTCACAGTCACCAA<br>AGTTGGGAAGTCACCCAACTTCGGGAACTTTGGGAAGTCACCCAAACTTACAGTCACCAAAATTGCTCTATTCTACTATGT<br>GACCTCAAAAGTGATTTGAAAGAAGGAACATCTGAGCTGGGCCCAAACCCTATTGCAATTTTATTGGGGCCAAAGAGAAC<br>TCCATGCTCCTGCCAAATCAAGGCAGTGTCAGCCTCAATAATTTCCCAGATAAAAATAAAAATCTGTGATACAATCAGAATG<br>TGAAAATTCTTATTTTGGAAGCAAATGTCATAACCAATGCAAGGGCTATCTCAATATTCATTCATTATGCAGTATTTTGAACT<br>GCAGTTGAAATGAATAAGAAGGAAAGGCAAACAACGAAGAGTCCAATTTCTCAATTTAGAAAAAGAGAAAAAAAAAGAAA<br>AGGGAGCACACAGGCACGGTGGCTCAAGCCTGTAATATCAGCACTTTGGCGGATCACTTGAGGTCAAGGAGTTCGAGAAA<br>AGAGAGCACCTAGAAGTTCAGCGCGGGATAATACTTAAGTAAATTATGACACCATCGTCTGTCATCTTGGGCCCATTCACTA<br>ACCCAAAGCTTTCAAAAGGGCTTTCTTAACCCTCACCTAGAATAGGCTTCCGCAGCCTTAATCCTTAGGGTGGCAGAATATC<br>AGGGACCCTGAGCATTCTTAAAAGATGTAGCTCGGGATGGGAAGTTCTTTTAATGACAAAGCAAATGAAGTTTCATTATGT<br>CGAGGAACTTTGAGGAAGTCACAGAATCCACGATTTAAAAATATATTTCCTATTATACACCCATACACACACACACACACCT<br>ACTTTCTAGAATAAAAACCAAAGCCATATGGGTCTGCTGCTGACTTTTTATATGTTGTAGAGTTATATCAAGTTATGTCAAGA<br>TGTTCAGTCACCTTGAAGAGGCTTTTATCAGAAAGGGGGACGCCTTTCTGATAAAGGTTAAGGGGTAACCTTAAGCTCTTA<br>CCCCTCTGAAGGTAAAATCAAGGTGCGTTCAGATGTTGGCTTGTTGTAAATTTCTTTTTTTATTAATAACATACTAAATGTGG<br>ATTTGCTTTAATCTTCGAAACTCTTCCCGGTGAAAATCTCATTTACAAGAAAACTGGACTGACATGTTTCACTTTCTGTTTCA<br>TTTCTATACACAGCTTTATTCCTAGGACACCAACACTAGATACCTAAACTGAAAGCTTCCGCCGATTTCACCGAAGGTCAGG<br>AAAGTCCAACGCCCGGCAAACTGGATTTGCTGCCTTGGGCAGAGGTGGGCGGGACCCCGCCTCCGGGCCTGGCGCAACG<br>CTGAGCAGCTGGCGCGTCCCGCGCGGCCCCAGTTCTGCGCAGCTTCCCGAGGCTCCGCACCAGCCGCGCTTCTGTCCGCC<br>TGCAGGTAGGGAGCGTTGTTCCTCCGCGGGTGCCCACGGCCCAGTATCTC |
| P2 (−1900 to +100 bp) | GAACAAAAACACGAATCCTCACATTACTAATACGCAAATCACTGAGCAGCAAGCTGAGCAAATACCCTCAATTCCCATCAG<br>CAACTTTAGAGAAAGGCAAATTCCGTTTGCCTCATTGATCATTTAGGTAGACCCTGAACACTGCTTTCATAAAACAAAAAC<br>AAAATACCCATCCCCAGTTTAAAAAATTATTCATAGATCATCCAGGCCATCTAGGAGGATATGATTTAATCCTGGCTACTTGG<br>TAAATTATTTGCCCAAGTTAACTCAGCTAGTTAGTGGTAGATGGCTCTGAAGCCAGTTGTTTTTTTTTGTTTTGTTTTTTGCA<br>GACCTCAAGAGTCATGATGAACTAGCAGATCATAAAGTTTATGCCCTGGGTCTTGACCATTTTTAGAAAAATAAAACATTAA<br>ATGAAAATATCAGAGGGCATTGCAGATAGTAGATCTAAGTATTTTTTCATGAAACTTGTTGTACATGTGTGTGTCATACACAG<br>ACTATATATATGCAGTACCTGTAAACTGTATTGCCACATAATGTCTATATTTTCCTAGAGGTCACAGTCACCAAAGTTGGGAA<br>GTCACCCAACTTCGGGAACTTTGGGAAGTCACCCAAACTTACAGTCACCAAAATTGCTCTATTCTACTATGTGACCTCAAA<br>AGTGATTTGAAAGAAGGAACATCTGAGCTGGGCCCAAACCCTATTGCAATTTTATTGGGGCCAAAGAGAACTCCATGCTCC<br>TGCCAAATCAAGGCAGTGTCAGCCTCAATAATTTCCCAGATAAAAATAAAAATCTGTGATACAATCAGAATGTGAAAATTCT<br>TATTTTGGAAGCAAATGTCATAACCAATGCAAGGGCTATCTCAATATTCATTCATTATGCAGTATTTTGAACTGCAGTTGAAA<br>TGAATAAGAAGGAAAGGCAAACAACGAAGAGTCCAATTTCTCAATTTAGAAAAAGAGAAAAAAAAAGAAAAGGGAGCAC<br>ACAGGCACGGTGGCTCAAGCCTGTAATATCAGCACTTTGGCGGATCACTTGAGGTCAAGGAGTTCGAGAAAAGAGAGCAC<br>CTAGAAGTTCAGCGCGGGATAATACTTAAGTAAATTATGACACCATCGTCTGTCATCTTGGGCCCATTCACTAACCCAAAGC<br>TTTCAAAAGGGCTTTCTTAACCCTCACCTAGAATAGGCTTCCGCAGCCTTAATCCTTAGGGTGGCAGAATATCAGGGACCCT<br>GAGCATTCTTAAAAGATGTAGCTCGGGATGGGAAGTTCTTTTAATGACAAAGCAAATGAAGTTTCATTATGTCGAGGAACT<br>TTGAGGAAGTCACAGAATCCACGATTTAAAAATATATTTCCTATTATACACCCATACACACACACACACACCTACTTTCTAGA<br>ATAAAAACCAAAGCCATATGGGTCTGCTGCTGACTTTTTATATGTTGTAGAGTTATATCAAGTTATGTCAAGATGTTCAGTCA<br>CCTTGAAGAGGCTTTTATCAGAAAGGGGGACGCCTTTCTGATAAAGGTTAAGGGGTAACCTTAAGCTCTTACCCCTCTGAA<br>GGTAAAATCAAGGTGCGTTCAGATGTTGGCTTGTTGTAAATTTCTTTTTTTATTAATAACATACTAAATGTGGATTTGCTTTAA<br>TCTTCGAAACTCTTCCCGGTGAAAATCTCATTTACAAGAAAACTGGACTGACATGTTTCACTTTCTGTTTCATTTCTATACA<br>CAGCTTTATTCCTAGGACACCAACACTAGATACCTAAACTGAAAGCTTCCGCCGATTTCACCGAAGGTCAGGAAAGTCCAA<br>CGCCCGGCAAACTGGATTTGCTGCCTTGGGCAGAGGTGGGCGGGACCCCGCCTCCGGGCCTGGCGCAACGCTGAGCAGC<br>TGGCGCGTCCCGCGCGGCCCCAGTTCTGCGCAGCTTCCCGAGGCTCCGCACCAGCCGCGCTTCTGTCCGCCTGCAGGTAG<br>GGAGCGTTGTTCCTCCGCGGGTGCCCACGGCCCAGTATCTC |
| P3 (−1400 to +100 bp) | ATATGCAGTACCTGTAAACTGTATTGCCACATAATGTCTATATTTTCCTAGAGGTCACAGTCACCAAAGTTGGGAAGTCACC<br>CAACTTCGGGAACTTTGGGAAGTCACCCAAACTTACAGTCACCAAAATTGCTCTATTCTACTATGTGACCTCAAAAGTGAT<br>TTGAAAGAAGGAACATCTGAGCTGGGCCCAAACCCTATTGCAATTTTATTGGGGCCAAAGAGAACTCCATGCTCCTGCCAA<br>ATCAAGGCAGTGTCAGCCTCAATAATTTCCCAGATAAAAATAAAAATCTGTGATACAATCAGAATGTGAAAATTCTTATTTT<br>GGAAGCAAATGTCATAACCAATGCAAGGGCTATCTCAATATTCATTCATTATGCAGTATTTTGAACTGCAGTTGAAATGAATA<br>AGAAGGAAAGGCAAACAACGAAGAGTCCAATTTCTCAATTTAGAAAAAGAGAAAAAAAAAGAAAAGGGAGCACACAGGC<br>ACGGTGGCTCAAGCCTGTAATATCAGCACTTTGGCGGATCACTTGAGGTCAAGGAGTTCGAGAAAAGAGAGCACCTAGAA<br>GTTCAGCGCGGGATAATACTTAAGTAAATTATGACACCATCGTCTGTCATCTTGGGCCCATTCACTAACCCAAAGCTTTCAA |

| | |
|---|---|
| | AAGGGCTTTCTTAACCCTCACCTAGAATAGGCTTCCGCAGCCTTAATCCTTAGGGTGGCAGAATATCAGGGACCCTGAGCA<br>TTCTTAAAAGATGTAGCTCGGGATGGGAAGTTCTTTTAATGACAAAGCAAATGAAGTTTCATTATGTCGAGGAACTTTGAG<br>GAAGTCACAGAATCCACGATTTAAAAATATATTTCCTATTATACACCCATACACACACACACACACCTACTTTCTAGAATAAA<br>AACCAAAGCCATATGGGTCTGCTGCTGACTTTTTATATGTTGTAGAGTTATATCAAGTTATGTCAAGATGTTCAGTCACCTTG<br>AAGAGGCTTTTATCAGAAAGGGGGACGCCTTTCTGATAAAGGTTAAGGGGTAACCTTAAGCTCTTACCCCTCTGAAGGTAA<br>AATCAAGGTGCGTTCAGATGTTGGCTTGTTGTAAATTTCTTTTTTTATTAATAACATACTAAATGTGGATTTGCTTTAATCTTC<br>GAAACTCTTCCCGGTGAAAATCTCATTTACAAGAAAACTGGACTGACATGTTTCACTTTCTGTTTCATTTCTATACACAGCT<br>TTATTCCTAGGACACCAACACTAGATACCTAAACTGAAAGCTTCCGCCGATTTCACCGAAGGTCAGGAAAGTCCAACGCCC<br>GGCAAACTGGATTTGCTGCCTTGGGCAGAGGTGGGCGGGACCCCGCCTCCGGGCCTGGCGCAACGCTGAGCAGCTGGCG<br>CGTCCCGCGCGGCCCCAGTTCTGCGCAGCTTCCCGAGGCTCCGCACCAGCCGCGCTTCTGTCCGCCTGCAGGTAGGGAGC<br>GTTGTTCCTCCGCGGGTGCCCACGGCCCAGTATCTC |
| P4 (−900 to +100 bp) | CCTGTAATATCAGCACTTTGGCGGATCACTTGAGGTCAAGGAGTTCGAGAAAAGAGAGCACCTAGAAGTTCAGCGCGGGA<br>TAATACTTAAGTAAATTATGACACCATCGTCTGTCATCTTGGGCCCATTCACTAACCCAAAGCTTTCAAAAGGGCTTTCTTA<br>ACCCTCACCTAGAATAGGCTTCCGCAGCCTTAATCCTTAGGGTGGCAGAATATCAGGGACCCTGAGCATTCTTAAAAGATGT<br>AGCTCGGGATGGGAAGTTCTTTTAATGACAAAGCAAATGAAGTTTCATTATGTCGAGGAACTTTGAGGAAGTCACAGAATC<br>CACGATTTAAAAATATATTTCCTATTATACACCCATACACACACACACACACCTACTTTCTAGAATAAAAACCAAAGCCATAT<br>GGGTCTGCTGCTGACTTTTTATATGTTGTAGAGTTATATCAAGTTATGTCAAGATGTTCAGTCACCTTGAAGAGGCTTTTATC<br>AGAAAGGGGGACGCCTTTCTGATAAAGGTTAAGGGGTAACCTTAAGCTCTTACCCCTCTGAAGGTAAAATCAAGGTGCGT<br>TCAGATGTTGGCTTGTTGTAAATTTCTTTTTTTATTAATAACATACTAAATGTGGATTTGCTTTAATCTTCGAAACTCTTCCCG<br>GTGAAAATCTCATTTACAAGAAAACTGGACTGACATGTTTCACTTTCTGTTTCATTTCTATACACAGCTTTATTCCTAGGACA<br>CCAACACTAGATACCTAAACTGAAAGCTTCCGCCGATTTCACCGAAGGTCAGGAAAGTCCAACGCCCGGCAAACTGGATT<br>TGCTGCCTTGGGCAGAGGTGGGCGGGACCCCGCCTCCGGGCCTGGCGCAACGCTGAGCAGCTGGCGCGTCCCGCGCGGC<br>CCCAGTTCTGCGCAGCTTCCCGAGGCTCCGCACCAGCCGCGCTTCTGTCCGCCTGCAGGTAGGGAGCGTTGTTCCTCCGC<br>GGGTGCCCACGGCCCAGTATCTC |
| P5 (-400 to +100 bp) | GACGCCTTTCTGATAAAGGTTAAGGGGTAACCTTAAGCTCTTACCCCTCTGAAGGTAAAATCAAGGTGCGTTCAGATGTTG<br>GCTTGTTGTAAATTTCTTTTTTTATTAATAACATACTAAATGTGGATTTGCTTTAATCTTCGAAACTCTTCCCGGTGAAAATCT<br>CATTTACAAGAAAACTGGACTGACATGTTTCACTTTCTGTTTCATTTCTATACACAGCTTTATTCCTAGGACACCAACACTAG<br>ATACCTAAACTGAAAGCTTCCGCCGATTTCACCGAAGGTCAGGAAAGTCCAACGCCCGGCAAACTGGATTTGCTGCCTTG<br>GGCAGAGGTGGGCGGGACCCCGCCTCCGGGCCTGGCGCAACGCTGAGCAGCTGGCGCGTCCCGCGCGGCCCCAGTTCTG<br>CGCAGCTTCCCGAGGCTCCGCACCAGCCGCGCTTCTGTCCGCCTGCAGGTAGGGAGCGTTGTTCCTCCGCGGGTGCCCAC<br>GGCCCAGTATCTC |

| Site | Sequences |
|---|---|
| -2354 to -2343 bp | TGCCCCCAAGTT |
| -2301 to-2290 bp | AACCCCAAGTCG |
| -2165 to -2137 bp | GCCCTGGCT |
| -1930 to -1919 bp | TGGCTGAAGGGT |
| -1530 to -1519 bp | TGCCCTGGGTCT |
| -1183 to -1175 bp | GCCAAAGAG |
| -923 to -912 bp | AGCACACAGGCA |
| -908 to -897 bp | TGGCTCAAGCCT |
| -766 to -756 bp | AACCCAAAGCT |
| -711 to -695 bp | GCCTTAATCCTTAGGGT |
| -498 to -490 bp | GCCATATGG |
| -371 to -361 bp | AACCTTAAGCT |
| -80 to -67 bp | GCTGCCTTGGGCAG |
| 54 to -33 bp | CCCCGCCTCCGGGCCTGGCGCA |
| -13 to +4 bp | TCCCGCGCGGCCCCAGTT |
| +14 to +24 bp | TTCCCGAGGCT |
| +47 to +58 bp | CGCCTGCAGGTA |
| +81 to +92 bp | TGCCCACGGCCC |
